# *Withania somnifera* (L.) Dunal (Ashwagandha) for the possible therapeutics and clinical management of SARS-CoV-2 infection: Plant-based drug discovery and targeted therapy

**DOI:** 10.3389/fcimb.2022.933824

**Published:** 2022-08-15

**Authors:** Manali Singh, Kuldeep Jayant, Dipti Singh, Shivani Bhutani, Nitesh Kumar Poddar, Anis Ahmad Chaudhary, Salah-Ud-Din Khan, Mohd Adnan, Arif Jamal Siddiqui, Md Imtaiyaz Hassan, Faez Iqbal Khan, Dakun Lai, Shahanavaj Khan

**Affiliations:** ^1^ Department of Biotechnology, Invertis University, Bareilly, Uttar Pradesh, India; ^2^ Department of Biochemistry, C.B.S.H, G.B Pant University of Agriculture and Technology, Pantnagar, Uttrakhand, India; ^3^ Department of Agricultural and Food Engineering, IIT Kharagpur, West Bengal, Kharagpur, India; ^4^ Department of Biosciences, Manipal University Jaipur, Jaipur, Rajasthan, India; ^5^ Department of Biology, College of Science, Imam Mohammad Ibn Saud Islamic University, Riyadh, Saudi Arabia; ^6^ Department of Biochemistry, College of Medicine, Imam Mohammad Ibn Saud Islamic University (IMSIU), Riyadh, Saudi Arabia; ^7^ Department of Biology, College of Science, University of Hail, Hail, Saudi Arabia; ^8^ Center for Interdisciplinary Research in Basic Sciences, Jamia Millia Islamia, New Delhi, India; ^9^ Department of Biological Sciences, School of Science, Xi’an Jiaotong-Liverpool University, Suzhou, China; ^10^ School of Electronic Science and Engineering, University of Electronic Science and Technology of China, Chengdu, China; ^11^ Department of Health Sciences, Novel Global Community Educational Foundation 7 Peterlee Place, Hebersham, NSW, Australia; ^12^ Department of Medical Lab Technology, Indian Institute of Health and Technology (IIHT), Deoband, Saharanpur, UP, India; ^13^ Department of Pharmaceutics, College of Pharmacy, King Saud University, Riyadh, Saudi Arabia

**Keywords:** SARS-CoV-2, ACE2 receptors, *Withania somnifera*, Ashwagandha, COVID-19, targeted therapy

## Abstract

Coronavirus disease 2019 (COVID-19) pandemic has killed huge populations throughout the world and acts as a high-risk factor for elderly and young immune-suppressed patients. There is a critical need to build up secure, reliable, and efficient drugs against to the infection of severe acute respiratory syndrome coronavirus 2 (SARS-CoV-2) virus. Bioactive compounds of Ashwagandha [*Withania somnifera (L.)* Dunal] may implicate as herbal medicine for the management and treatment of patients infected by SARS-CoV-2 infection. The aim of the current work is to update the knowledge of SARS-CoV-2 infection and information about the implication of various compounds of medicinal plant *Withania somnifera* with minimum side effects on the patients’ organs. The herbal medicine *Withania somnifera* has an excellent antiviral activity that could be implicated in the management and treatment of flu and flu-like diseases connected with SARS-CoV-2. The analysis was performed by systematically re-evaluating the published articles related to the infection of SARS-CoV-2 and the herbal medicine *Withania somnifera.* In the current review, we have provided the important information and data of various bioactive compounds of *Withania somnifera* such as Withanoside V, Withanone, Somniferine, and some other compounds, which can possibly help in the management and treatment of SARS-CoV-2 infection. *Withania somnifera* has proved its potential for maintaining immune homeostasis of the body, inflammation regulation, pro-inflammatory cytokines suppression, protection of multiple organs, anti-viral, anti-stress, and anti-hypertensive properties. Withanoside V has the potential to inhibit the main proteases (Mpro) of SARS-CoV-2. At present, synthetic adjuvant vaccines are used against COVID-19. Available information showed the antiviral activity in Withanoside V of *Withania somnifera*, which may explore as herbal medicine against to SARS-CoV-2 infection after standardization of parameters of drug development and formulation in near future.

## 1 Introduction

The infection of severe acute respiratory syndrome coronavirus 2 (SARS-CoV-2) has caused coronavirus disease 2019 (COVID-19). SARS-CoV-2 is a highly pathogenic virus that has spread very quickly throughout the globe. Many drugs and herbal compounds have been used for the management of this pandemic. Some important compounds of *Withania somnifera* may also have an antiviral activity, which may involve the management and treatment of SARS-CoV-2 infection. During the outbreak of COVID-19 in December 2019, Wuhan, the capital of the Hubei region and the most critical transportation center in China, witnessed harsh pneumonia-like symptoms. With its proximal origin on 31 December 2019, China informed the occurrence of an unknown disease to World Health Organization (WHO) and the Huanan seafood market was closed on 11 January 2020. The virus was recognized as a coronavirus on 7 January 2020, and it has more than 95% similarity with bat coronavirus and more than 70% homology related to SARS-CoV (World Health Organization, 2020). The first death was detected on 11 January 2020 after a daily rise in COVID-19 cases globally (Qin and Hernández, 2020).

A huge wave of infection of Omicron variant of SARS-CoV-2 is experienced throughout the world. The world is experiencing a huge wave of infection with the Omicron variant of SARS-CoV-2. The report of Institute for Health Metrics and Evaluation (IHME) on 17 January 2022 showed that about 125 million cases of Omicron infections observed in a single day throughout the world, which is more than 10-fold of the peak of the wave of Delta variant in April 2021 (IHME, 2022; Murray, 2022). Although the cases of Omicron has increased with compared with previous SARS-CoV-2 variants, the world infection-detection rate has decreased worldwide from 20% to 5% (IHME, 2022)

**Figure 1:**
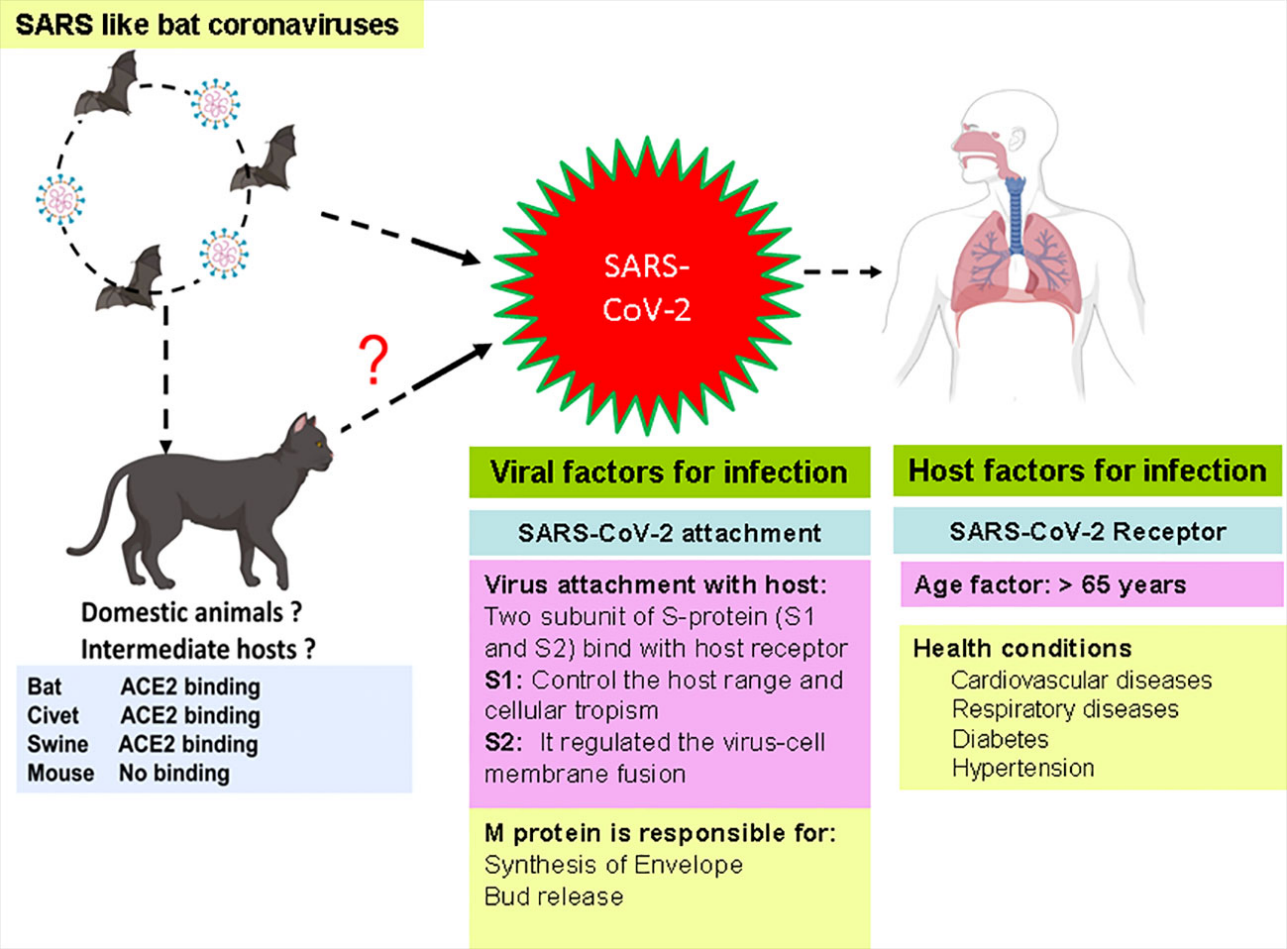
Factors connected with the infection of SARS-CoV-2 in human.

SARS-CoV-2 has been spread from animals to humans and from humans to humans and may cause respiratory, neurological, enteric, and renal symptoms (Swelum et al., 2020). The SARS-CoV-2 has several important proteins that help the virus in attachment, replication, and multiplication in host cells. Various factors such as host receptor, age of patients, and viral proteins have been connected with the transmission of SARS-CoV-2 in humans. These factors play an essential role in the transmission, infection, and multiplication of viruses. Recent studies showed the critical role of these factors in the infection of SARS-CoV-2 in the human and intermediate host (Zhao et al., 2020; Jackson et al., 2022) (**Figure 1**).

## 2 Genome and crucial proteins of SARS-CoV-2

The coronaviruses are a diverse group of enveloped positive-strand RNA viruses. The virus CoV-2 contains single-stranded positive senses ~30-kb RNA genomes that encode approximately 29 proteins. The 5′-end of the RNA is polyadenylated, whereas capping at the 3′-end increases the virus’s life span. The CoV-2 virus contains 6–11 open reading frames (ORFs), which encoded polyproteins having 9,680 amino acids (Guo et al., 2020). The ORF-1 encodes 16 nonstructural proteins (nsps), which comprises about 67% of the genome. The remaining ORFs encode structural proteins and other accessory proteins of the virus. The SARS-CoV-2 genome lacks the hemagglutinin-esterase gene. Nevertheless, it contains two flanking untranslated regions (UTRs) of 265 and 358 nucleotides long at 5′-end and 3′-end, respectively. The nsps comprises two viral cysteine proteases [papain-like protease (nsp3) and chymotrypsin-like protease], 3C-like, or main protease (nsp5), helicase (nsp13), and RNA-dependent RNA polymerase (nsp12), which involved in the process of replication and transcription of SARS-CoV-2 (Gao et al., 2020).

Four variants—Alpha CoV, Beta CoV, Gamma CoV, and Delta CoV of CoV-2—have been identified due to high rate of mutation in RNA virus. Recently, a new variant, Omicron, is also discovered in many countries. The possible adaptive mutations in the genome of SARS-CoV-2 may responsible to made it more pathogenic and hard for the development of vaccine and drug (Xu et al., 2020). This virus can circulate to several hosts by the loosely attached receptor binding domain with the virus (Raj et al., 2013). The virus entered into host cells that moderated to the host receptors through spike glycoprotein S; it is a major target for neutralization antibodies. Studies showed that the virus enters through the respiratory mucosa with the help of the receptor named ACE2 (angiotensin-converting enzyme 2) (Cheng and Shan, 2019; Balkrishna et al., 2020). S contains two functional subunits (S1 and S2 subunits) responsible for attaching the virus to the host cell receptor. According to structure, N- and C-terminal domains of the S1-fold are recommended as independent domains. Each of the N-terminal domains or the C-terminal domains can serve as a receptor-binding domain. CoV S protein is typically class 1 vital protein and proteases. The S protein of the virus is cleaved by the host proteases, and the subunits of the virus S protein remain present in a noncovalent form until the fusion of viral particle occurs. As a result, coronavirus entry into susceptible cells is a complicated process that needs the concerted action of receptor binding and proteolytic treating of the S protein to upgrade virus–cell fusion.

**Figure 2:**
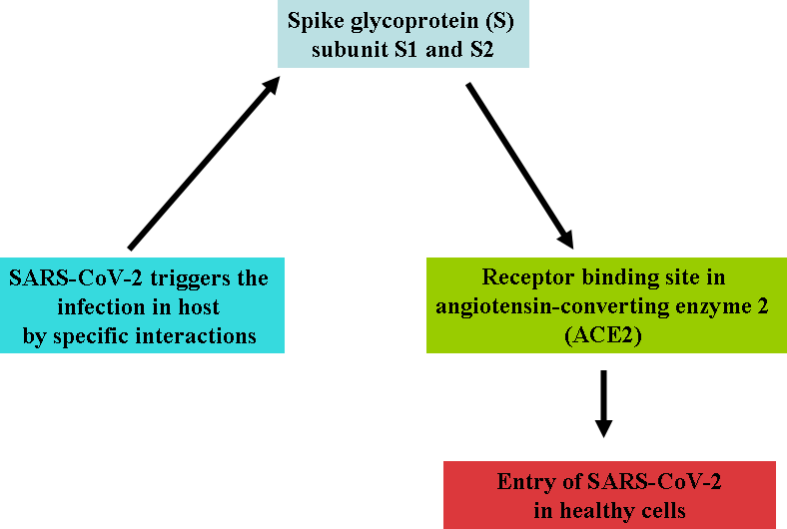
Attachment of SARS-CoV-2 with ACE2 binding site of the host.

Cleavage is essential for the establishment of the fusion property of S protein. The availability of these proteases on target cells highly determines whether viruses enter the host cells through endocytosis or plasma membrane. Proteases remain evasive, which promotes the entry of the virus into host cells. The study showed that SARS-CoV encoded S protein with 23 N-linked glycosylation sites (Rota et al., 2003). It is predicted that these 12 N-linked glycosylation sites play an essential role in attaching viruses to host cell receptors (Krokhin et al., 2003). ACE2 works like a port for coronavirus entry, which invades our infected cells and replicates. It is mainly found in our lungs and helps enter the virus into the host cells, but it also occurs in our GI tracts, heart, blood vessels, and muscles. The entry of SARS CoV-2 triggers the infection in healthy cells and may accelerate the death rates. SARS CoV-2 and another SARS-related coronavirus directly interact with ACE2 (**Figure 2**).

## 3 Mode of infection and multiplication of SARS-CoV-2

**Figure 3:**
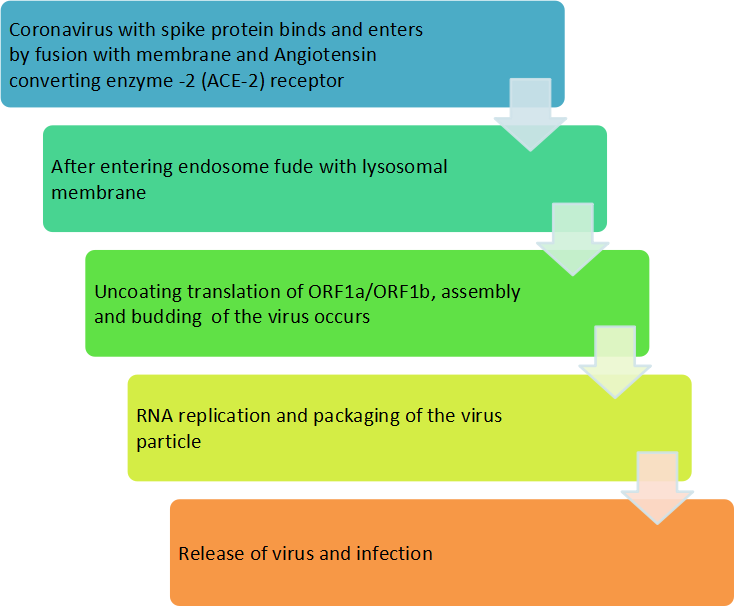
Schematic representation of transmission and infection of SARS-CoV-2 in the host cell.

The lung is the main target of SARS-CoV-2 infection, where the virus penetrates the infecting cells of host through binding with ACE2 (Seth et al., 2020). Although infection of SARS-CoV-2 commences in the proximal part of airways, more severe and occasionally fatal symptoms of the disease appeared in the alveolar type 2 cells of the distal lung (Mulay et al., 2021). The study also showed other symptoms such as fever, cold, gastrointestinal (GI) problem, nausea, vomiting and diarrhea upon hospital admission (Li et al., 2020). SARS-CoV-2 enters the respiratory tract and starts replicating in the epithelial cells of enteric tracts. The binding of SARS-CoV-2 with ACE2 induces the conformational changes in the S1 protein subunit, which helps in the exposure of S2 subunit. Depending on the entrance route chosen by the SARS-CoV-2, the S2 subunit is cleaved by different proteases and helps in the penetration of host cell. Fusion of SARS-CoV-2 with cell membrane of host cell generates a fusion pore by which RNA of the virus is delivered into the cytoplasm of infecting host cell for replication and multiplication of virus. The mode of transmission and infection is illustrated in flow chart (**Figure 3**).

**Figure 4:**
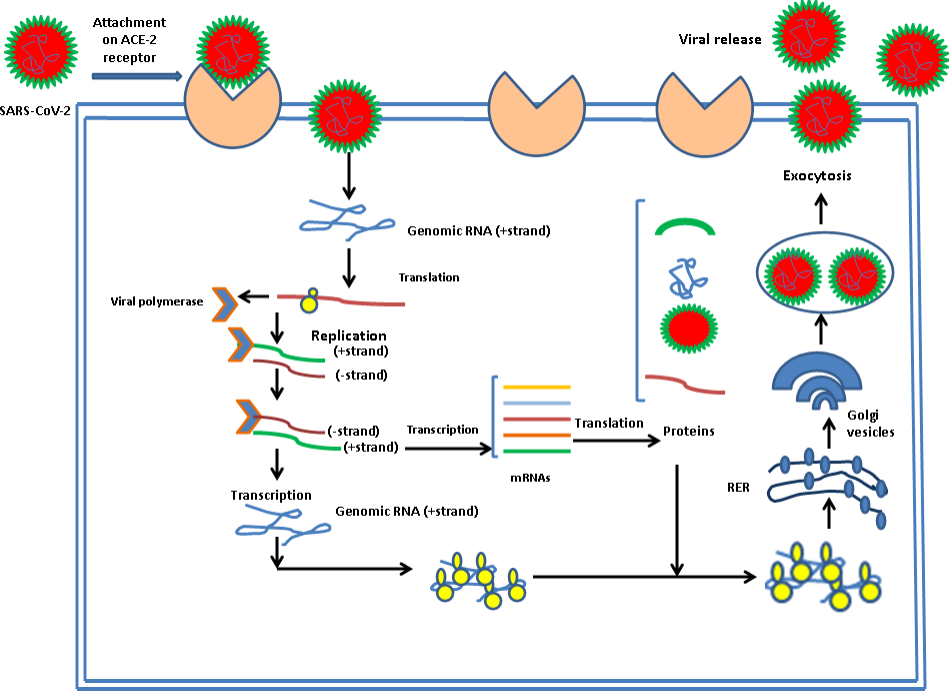
Entry, uncoating, and replication of SARS-CoV-2 in host.

There is a specific attachment of SARS-CoV-2 with the receptor of the infected host cell. A cellular protein is expressed on the surface of cells of different organs, known as ACE2 receptors for the attachment of SARS-CoV-2 particles. SARS-CoV-2 has a spike protein that binds with a particular receptor, facilitating the entry of the virus through fusion with membrane or ACE2 receptor. The virus genome is released in the cytoplasm of the host cell. The virus uses the host cell’s ribosomes to translate essential proteins required for the multiplication of the virus. Then, the transcription and translation of different ORF1a/ORF1b and others ORF occur. The assembly and budding of mature virions occur during RNA replication and packaging. The virus particles are released from the infected cell by exocytosis and further infects to healthy cells (**Figure 4**).

## 4 Clinical symptoms and diagnosis of SARS-CoV-2

The major symptoms of SARS-CoV-2 infection are fever, cough, throat sore, slight dyspnoea, fatigueness, conjunctivitis, headache, and GI problems (Yang et al., 2020; Pascarella et al., 2020). Symptoms of the disease may helpful for the identification of SARS-CoV-2 infection from other respiratory viral infections, specifically in community and urgent care setting programs where rapid diagnosis of samples may be inadequate. The documentation of symptoms should be helpful and supportive for the detection of new cases. The Centers for Disease Control and Prevention (CDC) of the United States identified three main symptoms of SARS-CoV-2, which include cough, fever, and shortness of breath (CDC, 2020). The symptoms list was increased as the pandemic of COVID-19 progressed, which include shortness of breath, myalgias, chills, sore throat, headache, chest pain, ageusia (the loss of taste), anosmia (the lose of smell), diarrhea, delirium, abdominal pain, skipped meals, and hoarse voice (Menni et al., 2020).

The symptoms of infection may confirm by performing reliable diagnosis including real‐time PCR (RT-PCR), computed tomography, and x-ray. Molecular diagnosis technique RT-PCR is implicated as a detection tool using samples of nasal swab, bronchoalveolar lavage or tracheal aspirate. Computed tomography and x-ray results are very imperative for both detection of SARC-CoV-2 infection and follow‐up the disease (Pascarella et al., 2020). A study suggested that diagnostic reliability and accuracy may be enhanced by combining clinical symptoms with the result of CT and RT-PCR. Prior to final conclusion, specimens of upper and/or lower respiratory tract should be re-diagnosed in the patients of negative RT-PCR report but has high suspicion or risk of infection (Lippi et al., 2020). This practice may minimize the possibility of error in diagnosis.

## 5 Strategies for the treatment of SARS-CoV-2

Various strategies have been implicated in the treatment and management of SARS-CoV-2 infection as follows.

### 5.1 Immunotherapeutic strategies to combat SARS-CoV-2

**Figure 5:**
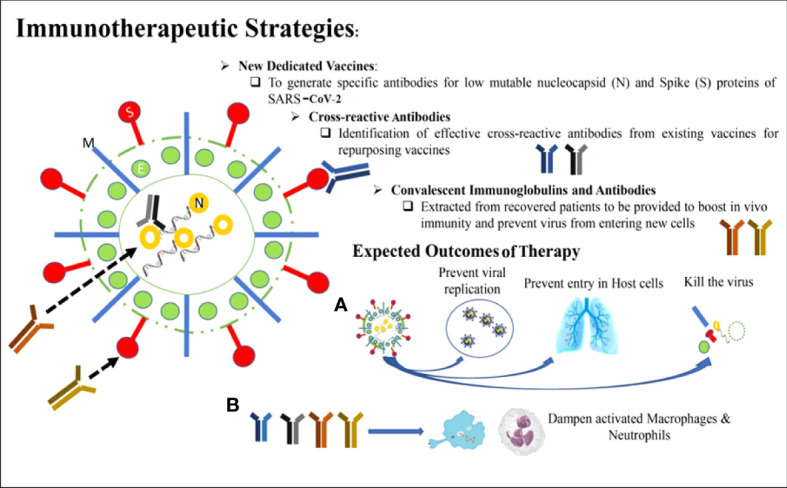
Immunotherapeutic strategies for the management and treatment of SARS-CoV-2.

The four vital targets of SARS-CoV-2 include N protein surrounding the viral RNA, E protein covering the viral envelope, M protein emerging from the cell membrane, and S protein interacting with the ACE2 receptor on the host cell. The IgG antibodies can neutralize less vulnerable viruses’ N and S protein and allow successful host immunity. These are also prospective targets for future vaccination approaches. The M and E proteins are frequently changed; hence, the potential of antibodies toward these proteins is not much more defensive during the infection of SARS-CoV-2. The cross-reactive antibody can provide a level of anti-SARS-CoV-2 protection that has developed during the treatment of measles and rubella viral infections through vaccination. Intravenous immunoglobin and different antibodies that are neutral in convalescent serum may block the entry of the virus into the host cells and reduce the hyper-inflammation (Gold et al., 2020) (**Figure 5**).

Baricitinib, a known Janus kinase (JAK) as a hindrance permitted for medical care of rheumatoid arthritis, might obstruct ACE2-mediated endocytosis (Richardson et al., 2020). The second one JAK as a hindrance, ruxolitinib, would be examined in a clinical investigation to remedy COVID-19 end of this year. Another way is to provide an excessive mass of a solvable form of ACE2, potentially decreasing virus entry into target host cells. This concept is being examined with APNO1; APEIRON is developed by rejoining the form of ACE2. It was recently in a clinical test. S protein is targeted by the monoclonal antibodies that obstruct the virus entry or fusion with the receptor of host cells. Nafamostat mesylate (Wang et al., 2020a) and camostat mesylate (Hoffmann et al., 2020). The protease inhibitor camostat mesylate inhibits the entry of SARS-CoV-2 in lung cells. INO-4800 DNA vaccine showed 100% immunogenic potential with outstanding safety and tolerability for the treatment of SARS-CoV-2 infection (Tebas et al., 2021). Although no specific clinical trials are performed to examine the potential of drugs against COVID-19 at this time of writing the review article, this combination therapy might be efficient because nafamostat mesylate hampers intravascular coagulopathy, to directly target the entry of virus in epithelial cells of the host. The treatment of a patient with combinational therapy might be effective as it has the potential to prevent the access of virus into the respiratory part (Hoffmann et al., 2020; Zhang et al., 2020). The inhibition of SMYD2 decreases the expression of TMPRSS2, which reduced the infection of SARS-CoV-2 by blocking the viral replication (Yu et al., 2022).

### 5.2 Inhibitors present in the host to prevent the spread of the virus

Our body reacts against the infection through various proteases like neutrophil elastase, mast cell tryptase, serine proteases, and others (Heutinck et al., 2010). The relation between the immune response and disease severity (immune-pathogenic reaction) led the idea to target proteases to control the threshold body’s response. Neutrophil elastase found in bone marrow is known to affect the levels of growth factor-alpha and stimulates mucus secretion (Nakamura et al., 1992). TMPRSS2, HAT, TTSPs, TMPRSS11a, Cathepsin B/L, and Factor Xa are transmembrane proteins that initiate spike protein priming, leading to ACE2 receptor–dependent viral entry (Bertram et al., 2011; Seth et al., 2020). It has been found that expression of chymase and tryptase of mast cells are different in the pulmonary and gastrointestinal tract. Because of the absence of protease inhibitors in lungs, uncontrolled elevated expression of granzymes leads to degradation of extracellular matrix and induces the induction of pro-inflammatory cytokines (Pejler et al., 2007). There are other potential host proteases that inhibit the SARS-CoV-2 virus that has been mentioned in **Table 1**.

**Table 1:** Potential host proteases that inhibit SARS-CoV-2 virus.

Inhibitors	Location	Mechanism Involved	References
Neutrophil elastase	Bone marrow	Affects the levels of growth factor alpha and stimulates mucus secretion	Nakamura et al. (1992); Roghanian and Sallenave (2008)
TMPRSS2 HAT TTSPs TMPRSS11a Cathepsin B/L Factor Xa	Transmembrane Histone protein Transmembrane Transmembrane Endosome Liver	Spike protein priming leading to ACE2 receptor–dependent viral entry.	Bertram et al. (2011); Seth et al. (2020)
Chymase Tryptase	Lining of intestine Connective tissue	Due to absence of protease inhibitors in lungs, uncontrolled elevated expression of granzymes lead to degradation of extracellular matrix and induce the induction of pro-inflammatory cytokines	Pejler et al. (2007)
Kallikrein-related peptidase 13	Chromosome	Involved in assisting human coronavirus entry by specific cleaving at S1/S2 site	Milewska et al. (2020)
KLK1 KLK5	Kidney, pancreas Epidermis	Involved in cleavage of hemagglutinin, enhancing the viral production	Leu et al. (2015); Magnen et al. (2017)
Proteinase 3	Neutrophils	Overexpression leads to uncontrolled degradation of extracellular matrix and inflammatory responses	Van der Geld et al. (2001)
Cathepsin C	Lysosome	Activation of several pro-inflammatory serine proteases	Méthot et al. (2007)
Granzymes A, B, H, K, and M Cathepsin G	NK cells Neutrophils	Causative agent of NK cells, T cells	Morice et al. (2007); Zhu et al. (2009); Jahrsdorfer et al. (2010)

The virus enters the host and can be prohibited by protease inhibitors because SARS-CoV-2 enters the host cell either by serine protease TMPRSS2 at the cell surface or by cysteine protease cathepsin L in the endosome. Hence, a combination of cathepsin L and serine protease TMPRSS2 inhibitors is efficient for inhibiting viral entry inside the host cell. Total inhibition is achieved by a couple of camostat mesylate and Cathepsin B/L inhibitor E-64d (Hoffmann et al., 2020). SPINT2, another gene-encoded protease inhibitor, targets TMPRSS2 by restricting the cleavage activation and viral growth (Magnen et al., 2017; Reid et al., 2017). Recently, a unique discovery of a new furin-like cleavage site in SARS-CoV-2 and a new furin-like protease recognition is in progress. Till now, it has been decided that the enzymatic activity of furin protease might be restricted by adding a decanoyl group at the N terminus and chloromethyl ketone (CMK) group to the C terminus of a polybasic cleavage motif (dec-RVKR-cmk) (Coutard et al., 2020).

### 5.3 Drugs and compounds used to combat SARS-CoV-2 infection

During the pandemic, various phytochemicals and repurposing drugs have been identified to understand their efficacy against different modes of action of viruses including SARS-CoV-2. Therapeutic drugs such as arbidol, hydroxychloroquine, chloroquine, and lopinavir have shown *in vitro* anti-coronaviral properties by preventing multiple pathways in the virus life cycle (Devaux et al., 2020; Yao et al., 2020; Zhou et al., 2020; Rai et al., 2022). Some of the important therapeutic interventions of repurposing drugs against coronaviruses are mentioned as follows:

1. Baricitinib (JAK1 and JAK2 inhibitor): It is an anti-inflammatory molecule approved by the FDA to cure RA, blocking the cytokine signaling to inhibit JAK (Richardson et al., 2020). Apart from inhibition, they regulate the receptor-mediated endocytosis by binding with the cyclin G-assisted kinase (Stebbing et al., 2020; Favalli et al., 2020). These inhibitors are used to disrupt receptor-mediated endocytosis and prevent viral entry into the host cell. However, side effects of the COVID-19 infection may limit the application of the JAK inhibitors in the infected patients (Sunzini et al., 2020; Akbarzadeh-Khiavi et al., 2022).
2. Remdesivir (nucleoside analog): Remdesivir is an analog of adenosine that inhibits the activity of viral RNA polymerase. It has two important targets including protease (Mpro) and RNA-dependent RNA polymerases (RdRp). It performed its action by interacting with RdRp through electrostatic force. On the other hand, it is interacted with protease Mpr by van der Waals interaction and formed Mpro–remdesivir complex. Thus, clinical trials show its efficacy against SARS-CoV-2 (Wang et al., 2020a; Elfiky, 2020). However, it shows hypersensitivity and infusion-related reactions. Remdesivir can cause elevated liver enzymes that can lead to liver injury (Thomas et al., 2020; Zampino et al., 2020).
3. Lopinavir/ritonavir and antiretrovirals (HIV protease inhibitor): An FDA-approved drug is useful against HIV; now, in the case of COVID-19, these are used for the hindrance of 3-chymotrypsin-like protease (Chu et al., 2004; de Wilde et al., 2014). They bind to the key enzyme, M^pro^, to suppress coronavirus activity. Safety concerns: risk of cardiac arrhythmias, the possibility of bradycardia, and should be carefully used with patients with hepatic disease.
4. Ivermectin (antiparasitic): They inhibit the duplication of SARS-CoV-2 by restraining the intracellular transport process followed by the virus to suppress the host antiviral response to inhibit the host alpha/beta-1 nuclear transport proteins (Wulan et al., 2015; Caly et al., 2020). It is harmful to patients with hepatic disease or asthma.
5. Actinomycin D (antibiotic): They activate the p53 molecule degraded by the SARS-CoV–encoded papain-like protease (PLpro) and suppress the interferon signaling. Thus, they enhance the p53’s expression by upregulating the interferon signaling/interferon production (Yuan et al., 2015; Krześniak et al., 2020).

#### 5.3.1 Side effects of drugs

With the massive use of repurposed remedies to cure COVID-19, a new enigmatic concern of antimicrobial resistance prevails. The resistance to various tetracycline antibiotics occurs because of the tet and otr genes acquisition. The increased resistance in microbes due to the chromosomal mutations or long constant exposure to the drugs has generated several other dysfunctions in the host’s body (Narendrakumar and Thomas, 2018; Narendrakumar et al., 2020; Singh et al., 2021). The central and peripheral nervous system gets affected by these drugs depending on the drug and its mode of action.

Protease inhibitors (Lopinavir, Ritonavir, and Darunavir) work against CYP450 by altering the blood plasma value of various psychotropic drugs. Being inherently neurotoxic, they show perioral paresthesias (25%) and peripheral paresthesias (7%) with depressive symptoms after the first month of treatment (Abers et al., 2014).

Corticosteroids alter the immune system by modulating hyperinflammation. Memory deficits and cognitive impairment occur in up to 90% of the patients. Approximately 50% of the patients feel delirium and mood changes after having a light dose for three months. Apart from these, mania and hypomania are also being observed (Fardet et al., 2007).

A monoclonal antibody named Tocilizumab, when used with IL-6 receptor at the critical point of COVID-19, triggers enormous growth of pro-inflammatory cytokines, leading to a cytokine storm. The heterogeneity of IL-6 and IL-1β increases the production of inflammatory cytokines (Mehta et al., 2020; WHO, 2020; Michot et al., 2020).

Patients treated with Bevacizumab head toward the vascular outburst that produces intrusion of inflammatory cells, due to which the already involved alveolar space becomes flooded with cells and other cytokines (Teuwen et al., 2020; Jacobi et al., 2020). The shoot of aerial levels of Vascular endothelial growth factor (VEGF) necessary for the vascular permeability causes serious issues [(USNLM, 2020) in serious patients with COVID-19 pneumonia].

The ACE2 effector drugs influence multiple vital organs through systemic inflammatory responses leading to disorders in the brain (encephalitis, necrotic hemorrhage, and epileptic seizures) (Carod Artal, 2020), disorders in the heart (cardiomyopathy and myocarditis) (Adão and Guzik, 2020), disorders in lungs (Varga et al., 2020), and disorders in the GI tract (Yang and Tu, 2020).

#### 5.3.2 Other variants of SARS-CoV-2

On 14 April 2022, the U.S. government SARS-CoV-2 Interagency Group (SIG) downgraded Delta from a variant of concern (VOC) to a variant being monitored (VBM) [National Center for Immunization and Respiratory Diseases (NCIRD), Division of Viral Diseases, 2020]. This new classification was based on significant and sustained reductions in its national and regional proportions over time. Evidence suggests that Delta variant does not currently pose a significant risk to public health in the United States. The SIG variant classification scheme defines four classes of SARS-CoV-2 variants (Public Health England, 2020)

- Variant being monitored (VBM): This might include many variants of SARS-CoV-2, which were found to infect people in different countries and regions with severe transmission rates.
- Variant of interest (VOI): These are those variations that are anticipated to have an impact on the virus’s ability to spread, the severity of the disease, immunological escape, and diagnostic or therapeutic escape. In addition, these could significantly affect community transmission, increasing the risk to public health globally.
- Variant of concern (VOC): These have been classified as the variants with a majority in epidemiological changes, causing the severe acute respiratory syndrome. These are known to be with increased virulence and lessen the effectiveness of the diagnostics and medical facilities. Some of them are listed as follows:

- Alpha (B.1.1.7 and Q lineages): This was the first time detected in the UK with a greater frequency of mutations. This variant has been found to spread faster at a very rapid rate of transmission (Bayarri-Olmos et al., 2021).
- Beta (B.1.351 and descendent lineages): This was for the first time detected in South Africa. This was the variant known to cause the second wave of the pandemic with higher transmission and severity levels (Roquebert et al., 2021). Later on, this variant spread to 23 states of the United States and 48 other countries. Its mutations were notable due to its enhanced ability to bind to ACE-3 receptor protein which brought serious concern internationally (Bayarri-Olmos et al., 2021).
- Gamma (P.1 and descendent lineages): SARS-CoV-2 lineage P.1 was first discovered in Brazil in November 2020. It comes from the lineage B.1.1.28. It has three problematic mutations—N501Y, E484K, and K417T—as well as 17 distinctive amino acid alterations, 10 of which are in the spike protein. With the same capacity to infect both adults and old persons, it demonstrated 2.2 times increased transmissibility. It was classified as a VOC by WHO on 11 January 2021. WHO gave it the name Gamma on 31 May 2021.
- Delta (B.1.617.2 and AY lineages): SARS-CoV-2 lineage B.1.617.2 was first discovered in India in October 2020. It was designated as a VOC on 11 May 2021, and, on 31 May 2021, it was given the name Delta. It carries the L452R, T478K, and P681R mutations and can spread approximately two times as quickly as the Alpha form. The “Delta plus” or “AY.1” variant is a further mutation of the extremely contagious Delta variant. Because of the newly added variant’s low occurrence in India, it is not yet a VOC.
- Epsilon (B.1.427 and B.1.429): SARS-CoV-2 of lineages B.1.427 and B.1.429 was first detected in the United States in March 2020. I4205V and D1183Y in the ORF1ab-gene, as well as S13I, W152C, and L452R in the spike protein’s S-gene, are two of the five unique mutations that characterize it. The WHO identified it as a VOI on 05 March 2021, and, on May 31, 2021, it was given the name Epsilon.
- Eta (B.1.525): In December 2020, reports of SARS-CoV-2 lineage B.1.525 were made in numerous nations. It is a variant under investigation according to Public Health England (VUI-21FEB-03). The E484K mutation and the novel F888L mutation set Eta apart from all previous forms. The WHO identified it as a VOI on 17 March 2021, and, on May 31, 2021, it was given the name Eta.
- Iota (B.1.526): In November 2020, the SARS-CoV-2 lineage B.1.526 was first discovered in the United States. Initial levels were rather high in some areas, but in the spring of 2021, the more transmissible Alpha version outcompeted it. The WHO identified it as a VOI on 24 March 2021, and, on May 31, 2021, it was given the name Iota.
- Kappa (B.1.617.1): SARS-CoV-2 lineage B.1.617.1 was first l discovered in India in December 2020. Public Health England recognized it as a variant under investigation on 01 April 2021 (VUI-21APR-01). On 04 April 2021, WHO classified it as a VOI, and, on May 31, 2021, it was given the name Kappa.
- 1.617.3: A recently described SARS-CoV-2 variant under examination was first discovered in October 2020 in India and is also known as G/452R.V3 and is now designated by WHO with the Greek letters. Three sublineages identified as B.1.617.1 (), B.1.617.2 (), and B.1.617.3 as of May 2021 are being investigated for their potential effects on the ongoing epidemic. There are 13 amino acid alterations in this variation, three of which in the spike protein—E484Q, L452R, and P681R—are currently of considerable concern (Pascarella et al., 2021)
- Mu (B.1.621 and B.1.621.1): SARS-CoV-2 lineage B.1.621 was first detected in Colombia in January 2021. On 30 August 2021, the WHO classified it as a VOI and gave it the moniker Mu.
- Zeta (P.2): SARS-CoV-2 lineage P.2 was first discovered in Brazil in April 2020. The WHO identified it as a VOI on 17 March 2021, and, on May 31, 2021, it was given the name Zeta.
- Omicron (B.1.1.529, BA.1, BA.1.1, BA.2, BA.3, BA.4, and BA.5 lineages): In November 2021, South Africa received the first report of the B.1.1.529 lineage. On 26 November 2021, the WHO gave it the name Omicron and classified it as a VOC. According to reports, the Delta form of COVID-19, the earlier COVID-19 variation, is less deadly than the most recent SARS-CoV-2 variant. Omicron can spread swiftly, is infectious, and is not immune to the current vaccination.

IV. Variant of high consequence (VOHC): To date, no variants of high consequence have been identified in the United States.

### 5.4 Current status of vaccine and their issues

As of 08 April 2022, WHO has evaluated that the following vaccines against COVID-19 have met the necessary criteria for safety and efficacy: AstraZeneca/Oxford vaccine, Johnson and Johnson, Moderna, Pfizer/BioNTech, Sinopharm, Sinovac, COVAXIN, Covovax, Nuvaxovid, and CanSino. However, there were some adverse reactions reported in people due to prior allergies (Sumito et al., 2021). Although *Withania somnifera* cannot fully eradicate SARS-CoV-2, studies have shown that co-administration of Ashwagandha with the COVISHIELD™ vaccine enhanced the antibody titers, particularly after the first dose of the vaccine (Chopra et al., 2022).

## 6 Herbal medicine

Various herbal medicines have been used to treat and manage diseases, including microbial infections such as viral, bacterial, and fungal diseases. Extracts of the herbal plants have been found as broad-spectrum anti-coronaviral drugs shown with less or no side effects (Sharma et al., 2021). Wijayasinghe et al. have shown that most of the anti-coronaviral phytochemicals from different herbal plants are not only effective against viral proteases but also affected the other mechanisms such as viral protein synthesis, viral entry, and viral replication (Wijayasinghe et al., 2021). Therefore, to accomplish the strategy, *Withania somnifera* is one of the potential herbal medicines that can be used against various types of infections.

### 6.1 *Withania somnifera* works as a potent drug in combating COVID-19

WHO’s COVID-19 management techniques include infection prevention, detection of the cases, and supportive care. *Withania somnifera* belongs to the *Solanaceae* family that grows to 35–75 cm in the drier parts of India (Narinderpal et al., 2013). All aspects of this therapeutically important plant are used for treating osteoarthritis, gout, neurodegenerative diseases, and many more (Dar and MuzamilAhmad., 2020). The chemical constituents of the *Withania somnifera* genus include withanolides (class of naturally occurring C28 steroidal lactone triterpenoids along with, steroidal lactones, alkaloids, tropine, and cuscohygrine) (Singh et al., 2016). The anti-inflammatory and analgesic attributes are supported by the inhibition property of cyclo-oxygenase-2 (Ryu et al., 2010).

Withanolides contribute to the pharmacological benefits of *Withania somnifera*. A bioactive steroidal lactone withaferin A reduces the secretion of various pro-inflammatory cytokines, for example, TNFα, IL-6, IL-8, and IL-18 (Straughn and Kakar, 2019), whereas withanone blocks the SARS-CoV entries by lowering the electrostatic component of ACE2-RBD complex (Mi-Sun et al., 2008), and it hinders the ventures and regulation of cell surface receptor protein TMPRSS2 and viral replicative protease M^pro^ (Dhanjal et al., 2021; Markus et al., 2020). *In silico* studies represent that *Withania somnifera* can repress the replication of the COVID-19 virus through its capability to adjust T-cell separation NK-cell cytotoxicity (Maurya and Sharma, 2020). Several withanolides cause downregulation of viral envelope (E-gene) expression and nucleoplasmic sequences (N-gene). The organ preservative outcomes of *Withania somnifera* are used to reduce systemic inflammation, which protects the severity of inflammation-induced organ damage. Apart from these anti-viral actions, the Ministry of AYUSH has also confirmed the role of *Withania somnifera* for the maintenance of mental health, which may very helpful for the management and treatment of COVID-19 (Chopra et al., 2021).

*Withania somnifera* has showed many benefits such as 1) maintaining the immune balance of the body, 2) regulation of the inflammation, 3) suppression of pro-inflammatory cytokines, 4) protection of multiple organs, and 5) anti-viral, anti-stress, and anti-hypertensive properties. The plant’s phytochemicals, in combination with drugs or other clinical treatments, can be used in therapeutic purpose including management of infection of the SARS-CoV-2. Several docking and simulation (under clinical trial) experiments have removed the dust from the idea of obstructing the translation of viral protein. Some of the important bioactive metabolites extracted from *Withania somnifera* could be promising in combating SARS-CoV-2 (Trivedi and Choudhrey, 2011). *In silico* studies revealed that the methanolic extract of *Withania somnifera* containing Anaferine, Cuscohygrine, and Hygrine has been found to that bind at the specified binding site for the agonists of α7 nAChR, thus avoiding dysregulation of the NCS (Nicotinic Cholinergic System) and moderating the symptoms and clinical manifestations of COVID-19 (Cardenas et al., 2012). Whereas Tropine, Choline, Withanolide D, and Withanisomniferol C were found to Inhibit PLpro and 3CLpro and bind to spike (S) protein (Chatterjee et al., 2010; Tomar et al., 2019). Mesoanaferine, Withanolide O, Withanolide P, Withanolide G, Withanolide F, Withanoside IV, Withanolide D, β-sitosterol, and Somniwithanolide have shown to inhibit main protease (M^pro^) of SARS-CoV-2. Apart from these, there are other several potent compounds that may be effective against the infection of SARS-CoV-2, which are also mentioned in **Table 2**.

**Table 2:** Important bioactive metabolites extracted from *Withania somnifera* may be used for the management of COVID-19.

S.No.	Phytochemicals	Pubchem Id	Binding energy (kcal/mol)	Molecular weight (g/mol)	Part of Plant used	Structure of Phytochemical	Nature	References
1.	Tropine	449293	-2.54	141.21	Leaves	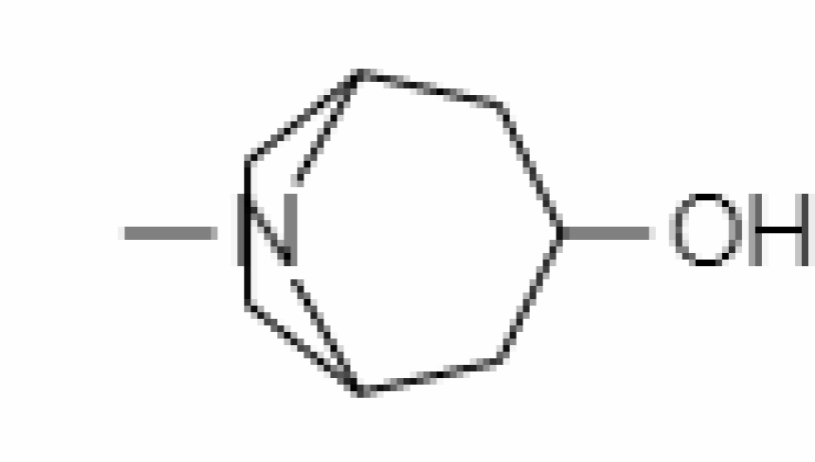	Methanolic	Chatterjee et al. (2010); Tomar et al. (2019)
2.	Mesoanaferine	443143	-3.42	224.34	Leaves	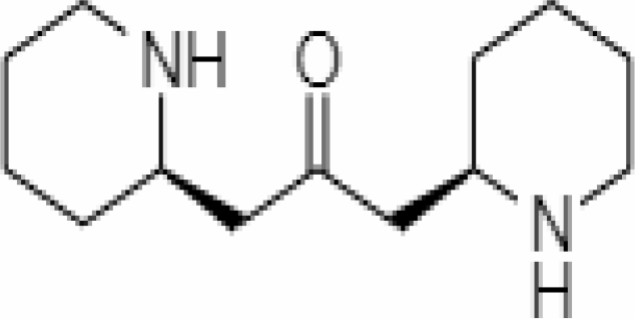	Methanolic	Cardenas et al. (2012); Kumar and Singh (2020)
3.	Choline	305		104.17	Leaves	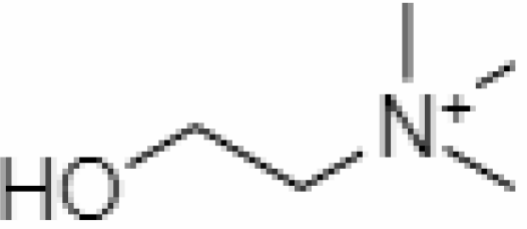	Methanolic	Ahlawat et al. (2017)
4.	Withanolide D	23266147	-8.9	470.6	Leaves	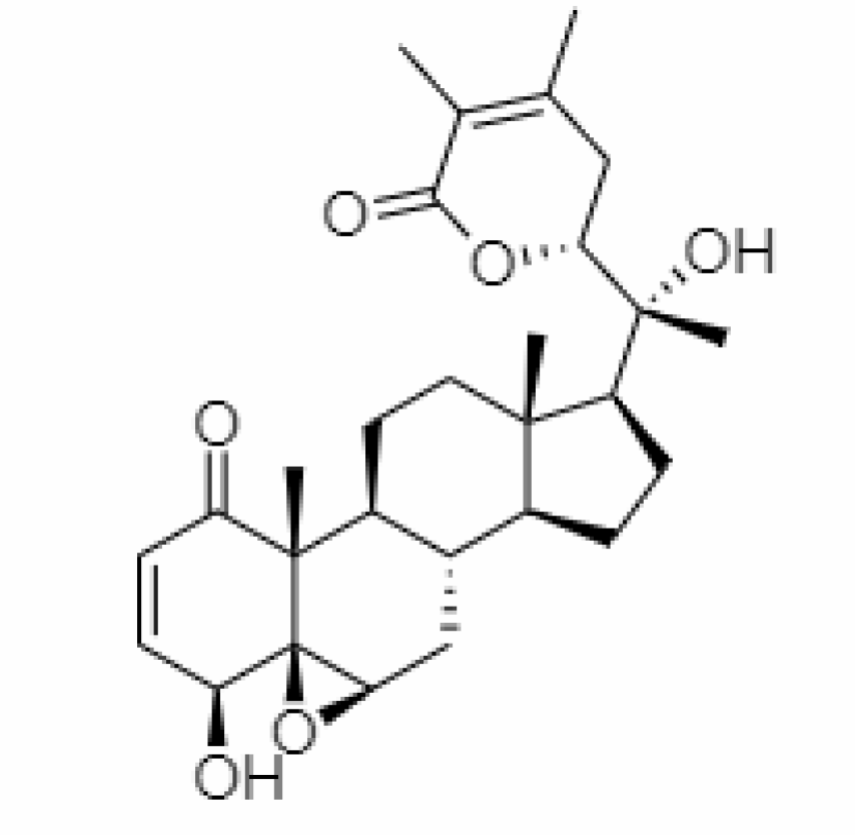	Methanolic	Ben Bakrim et al., 2018
5.	Withanolide O	23266146	-7.8	454.6	Leaves	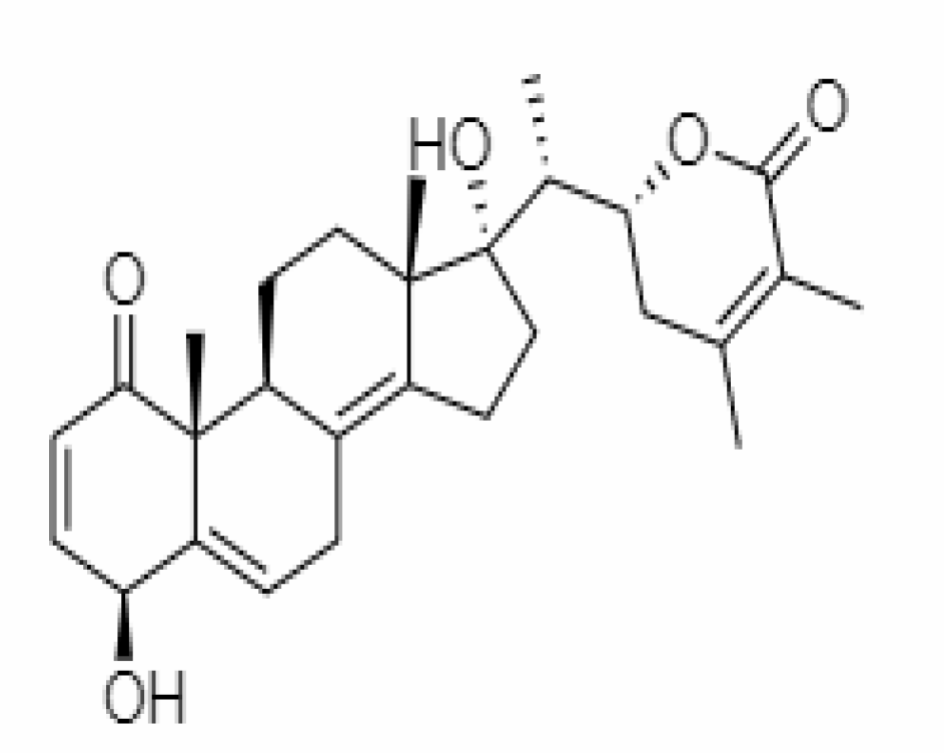	Methanolic	Khanal et al. (2020)
6.	Withanolide P	21679034	-7.7	468.6	Leaves	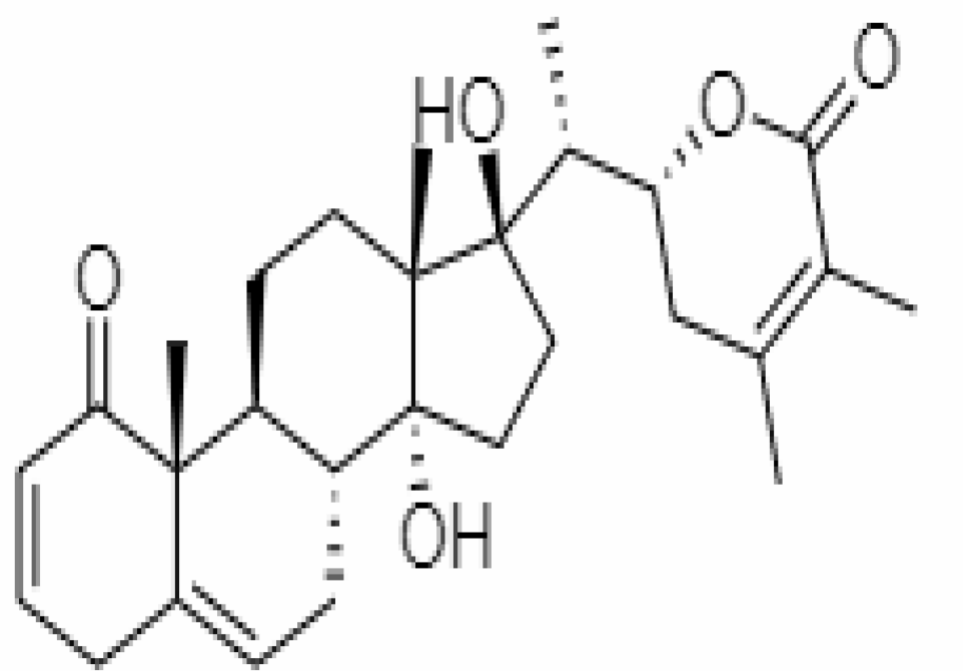	Methanolic	Khanal et al. (2020)
7.	Withanolide G	21679023	-9.00	470.6	Leaves	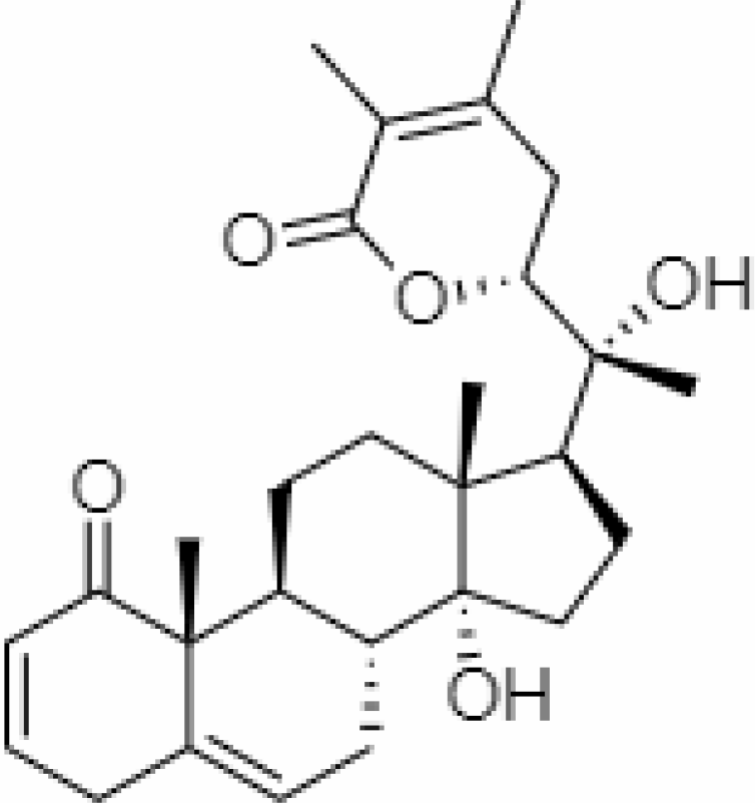	Alcoholic	Mousavi et al. (2021)
8.	Withanolide M	25090669	-9.1	652.8	Leaves	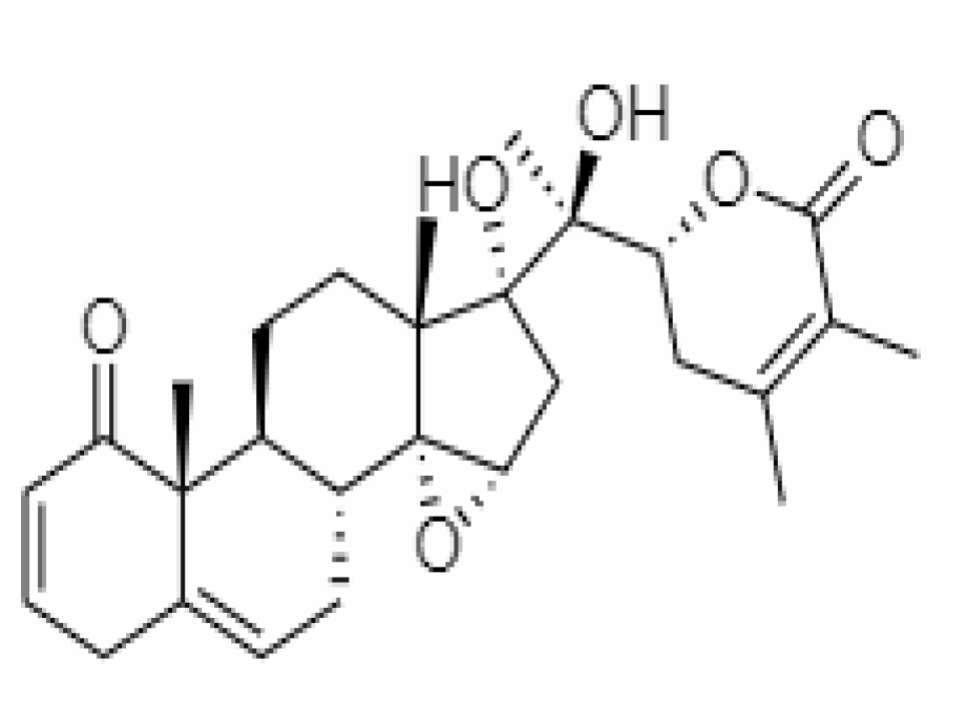	Alcoholic	Khanal et al. (2020)
9.	Withanolide F	44562999	-7.8	488.6	Leaves	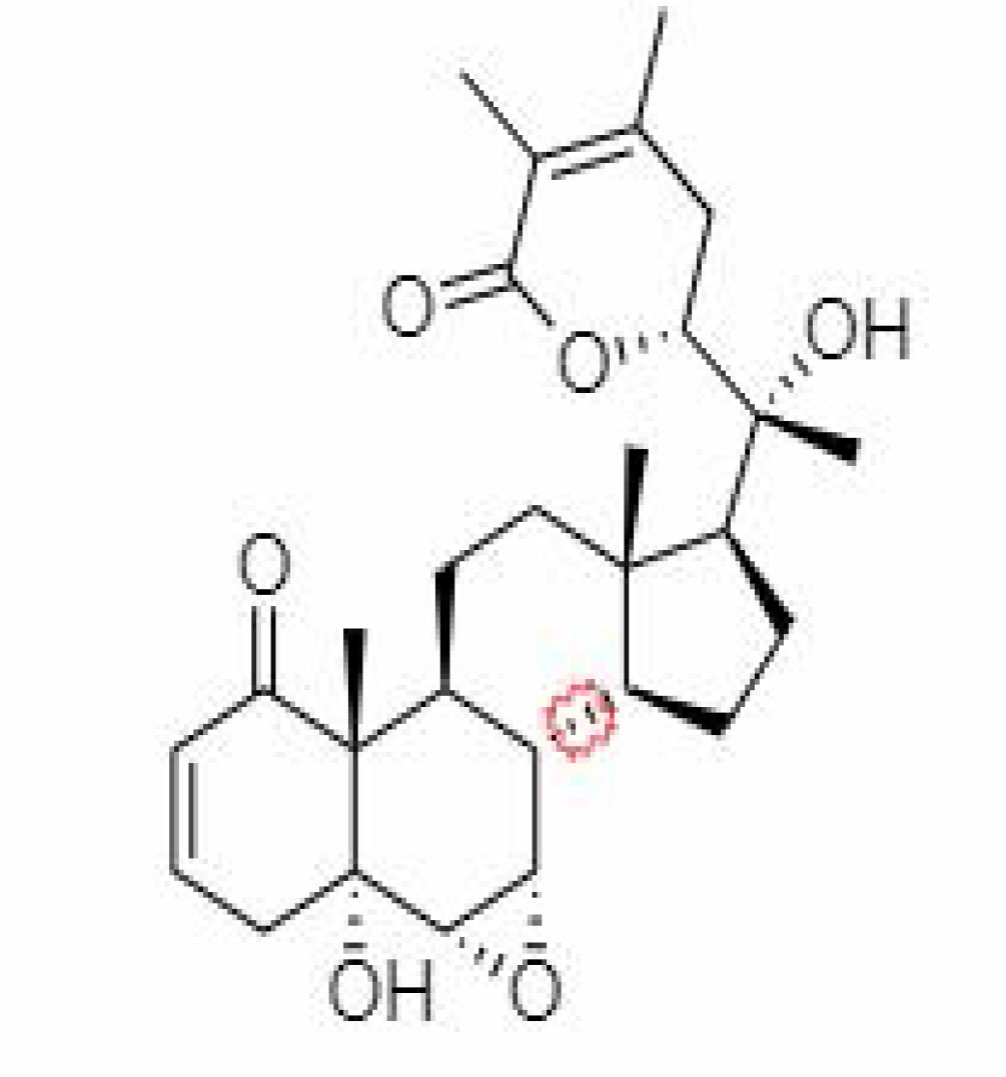	Alcoholic	Khanal et al. (2020)
10.	Withanoside IV	71312551	-11.02	782.9	Leaves	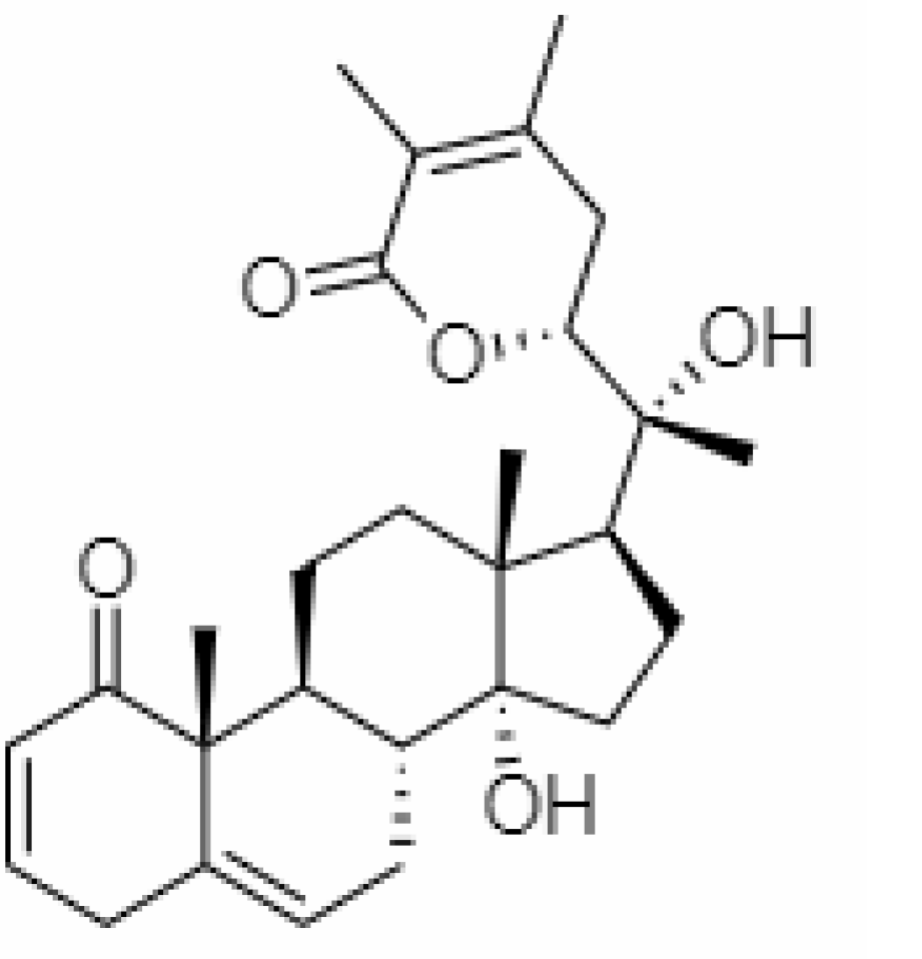	Butanol	Ali et al. (2022)
11.	Withanoside VI	91827019	8.083	782.9	Leaves	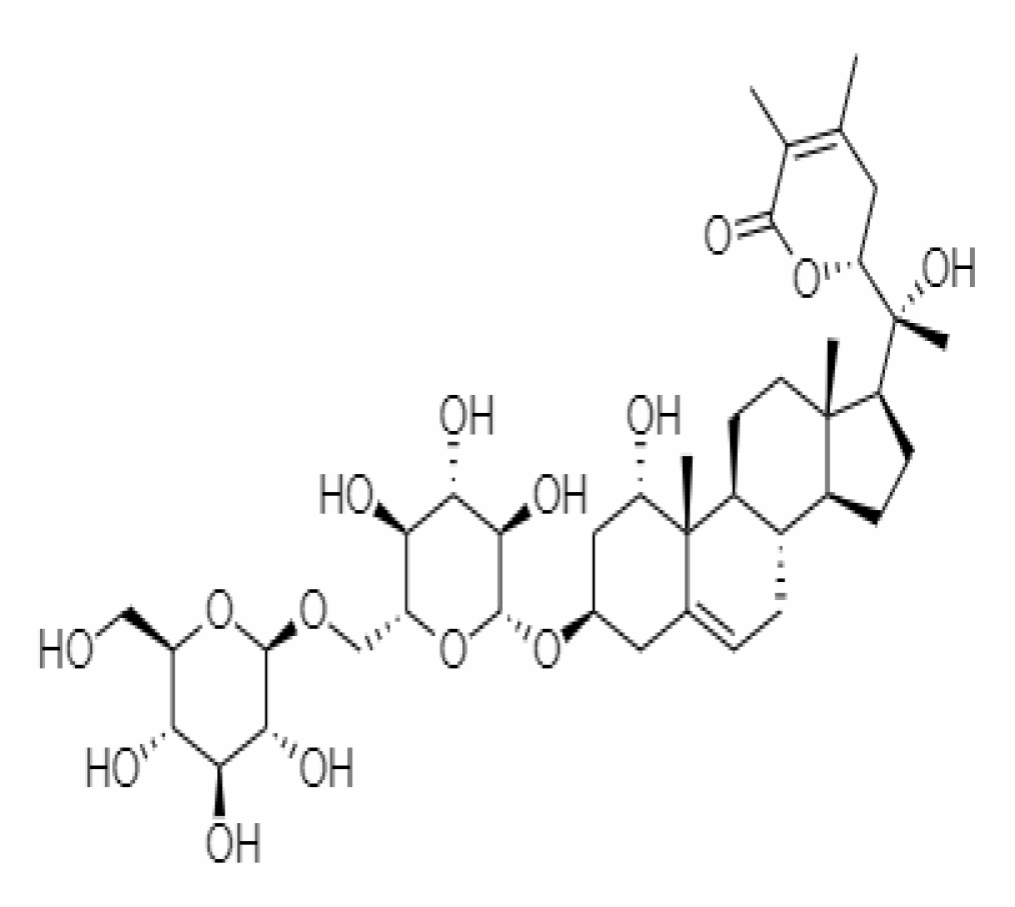	Methanolic	Patel et al. (2021)
12.	Withaferin A	265237	-2.85	470.6	Leaves and Roots	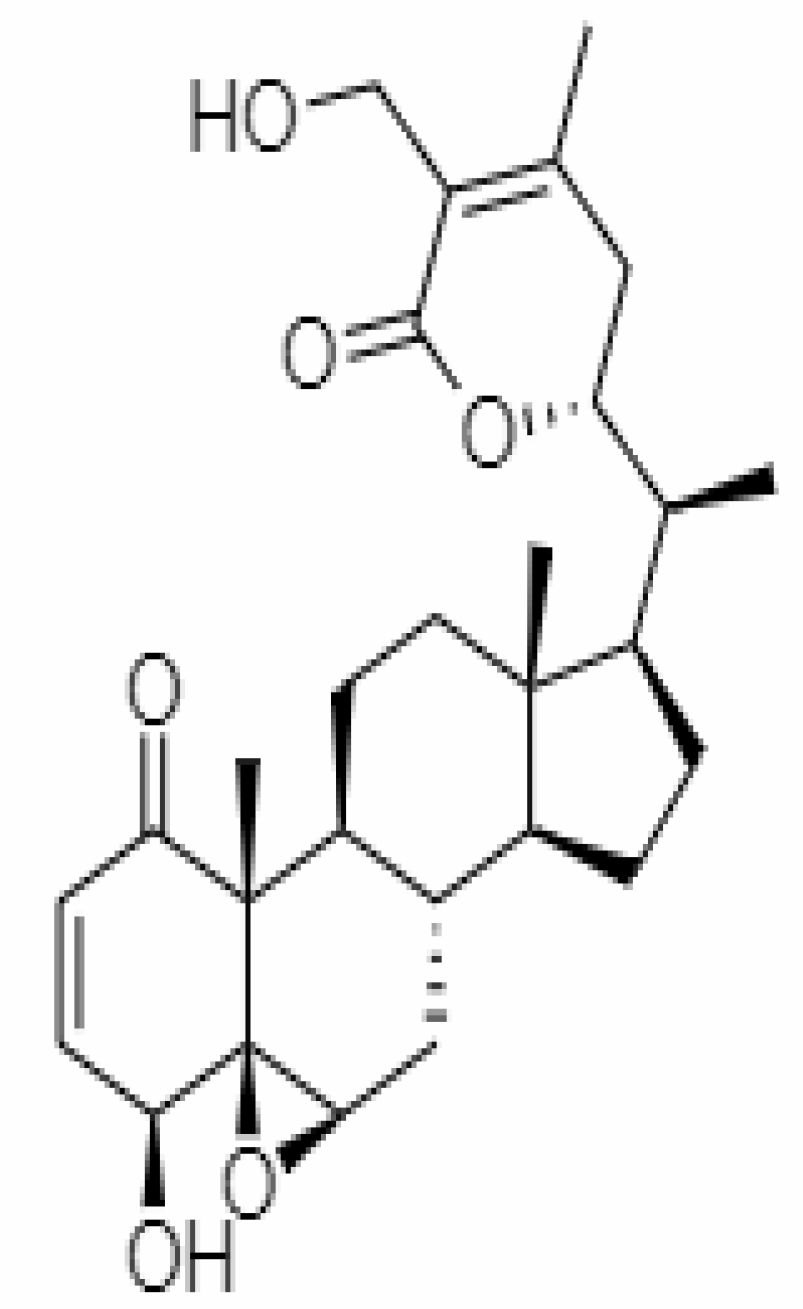	Methanolic	Balkrishna et al. (2020); Kumar and Singh (2020); Mohan et al. (2016); Kumar et al. (2022); Pandit and Latha (2020)
13.	Withanolide D	118701104	-5.55	470.6	Leaves and Roots	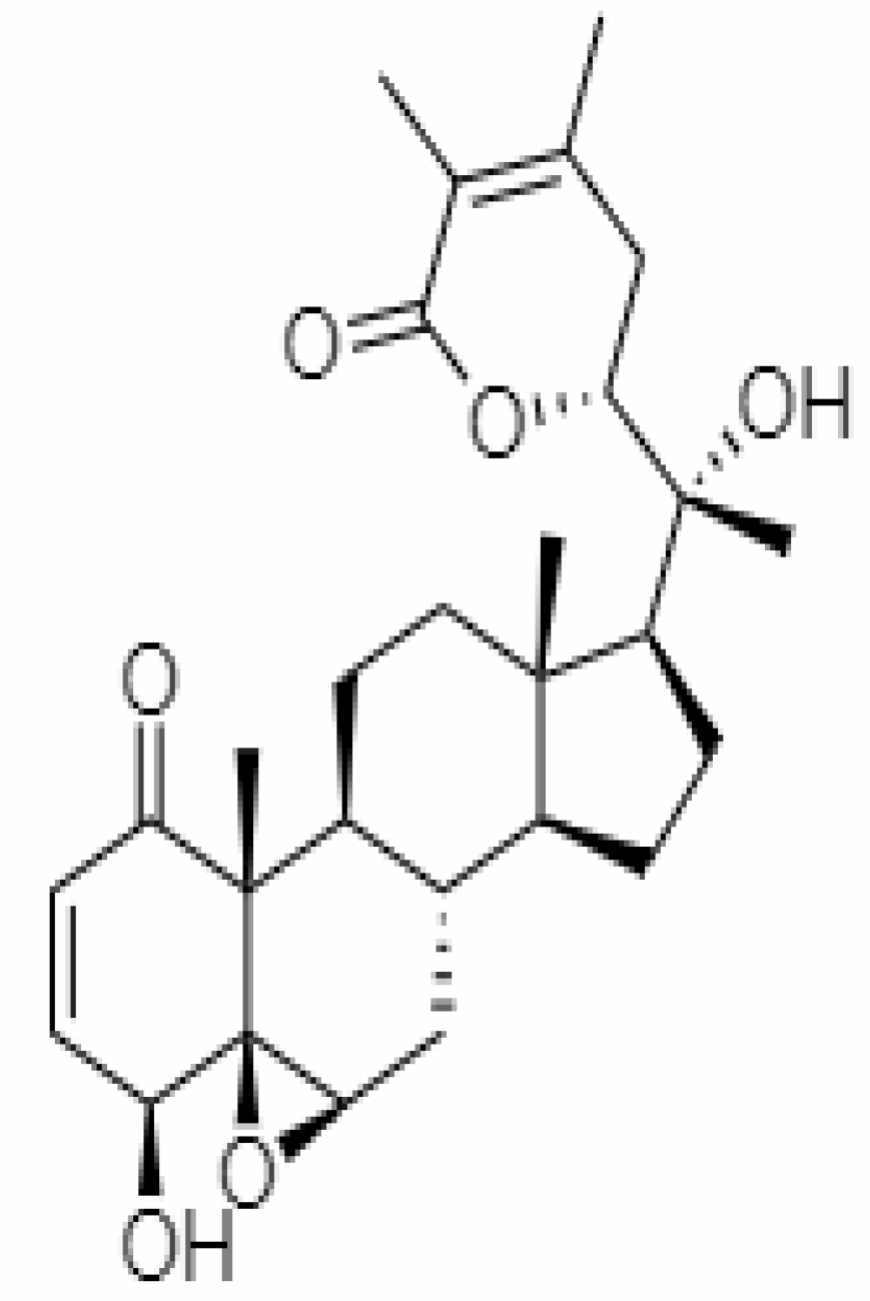	Methanolic	Khanal et al. (2020)
14.	Withanolide A	11294368	-5.26	470.6	Leaves and Roots	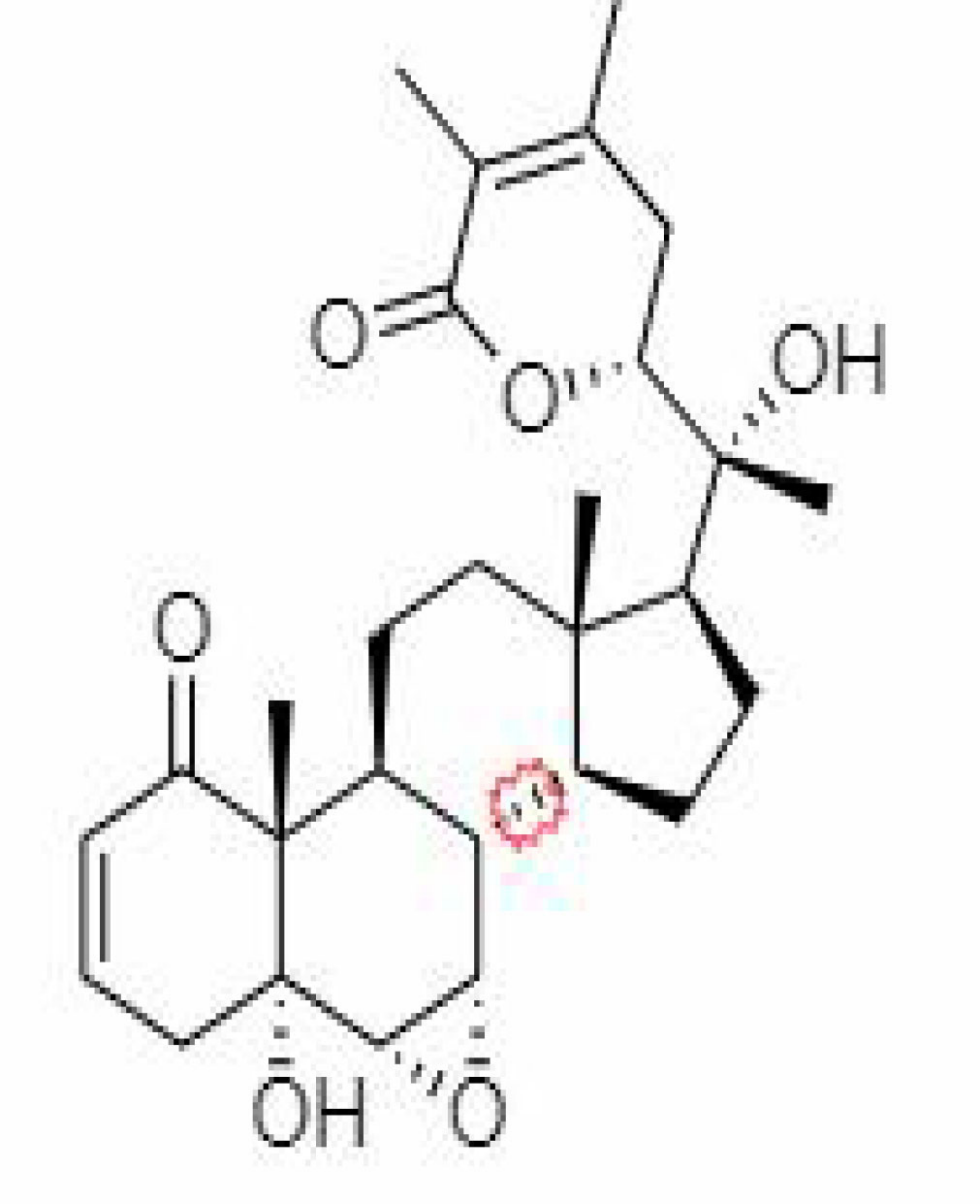	Methanolic	Kour et al. (2009); Soman et al. (2013); Pandit and Latha (2020)
15.	Withanone	21679027	-6.14	470.6	Leaves and Roots	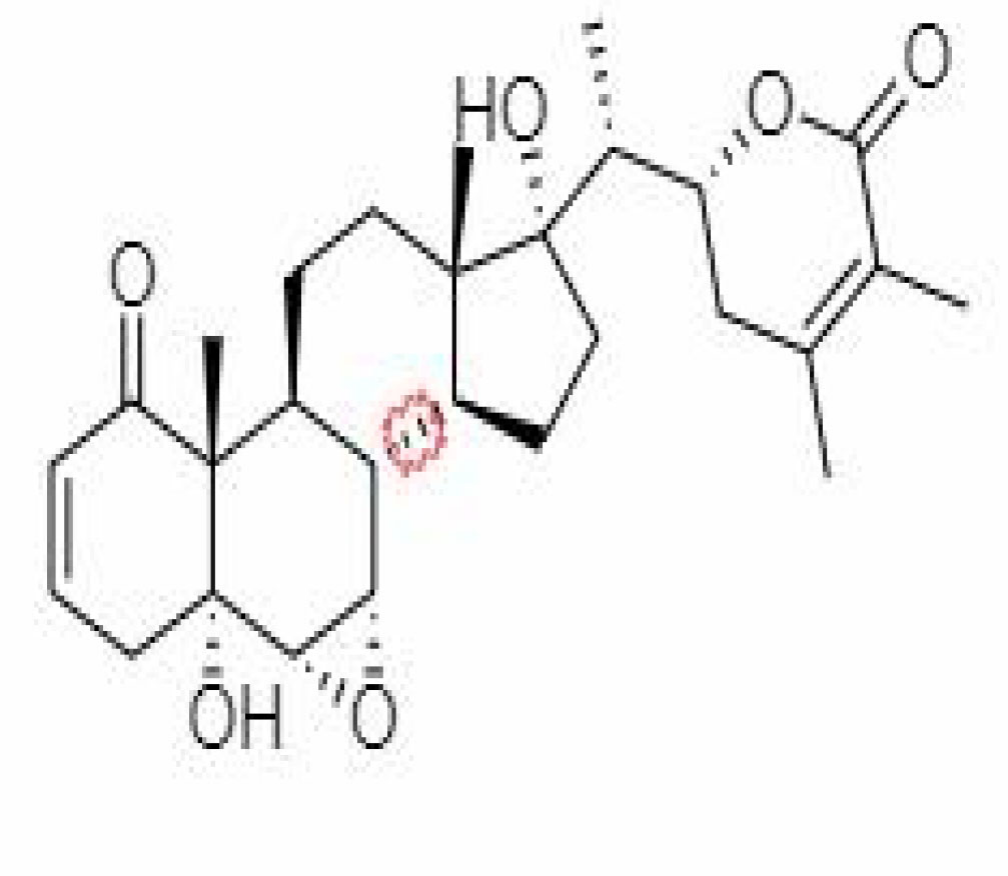	Methanolic	Wadegaonkar and Wadegaonkar (2013); Dar et al. (2017); Kumar and Singh (2020); Balkrishna et al. (2020)
16.	27-hydroxy withanolide B	14236711	-5.23	454.6	Leaves and Roots	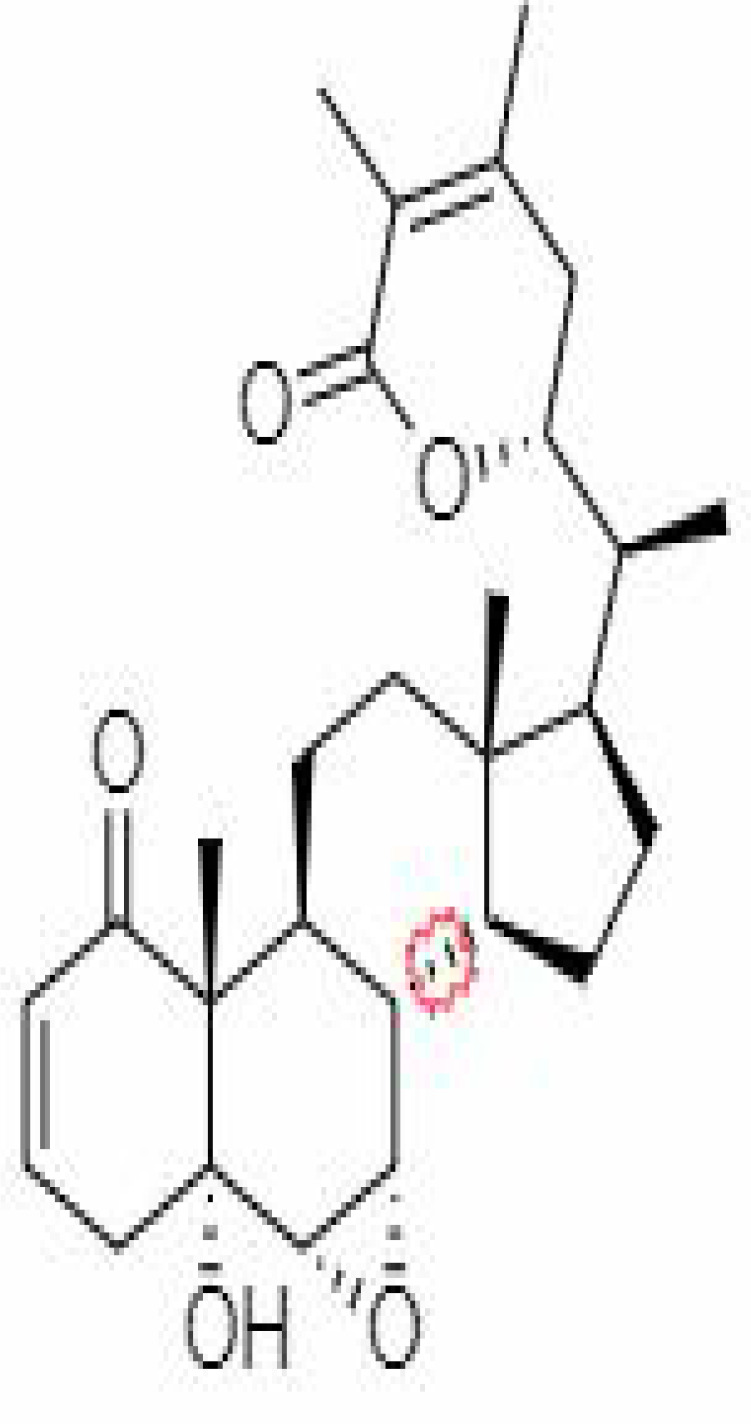	Methanolic	Turrini et al. (2016); Balkrishna et al. (2021)
17.	Pseudowithanine	10955717	-3.34	241.33	Leaves and Roots	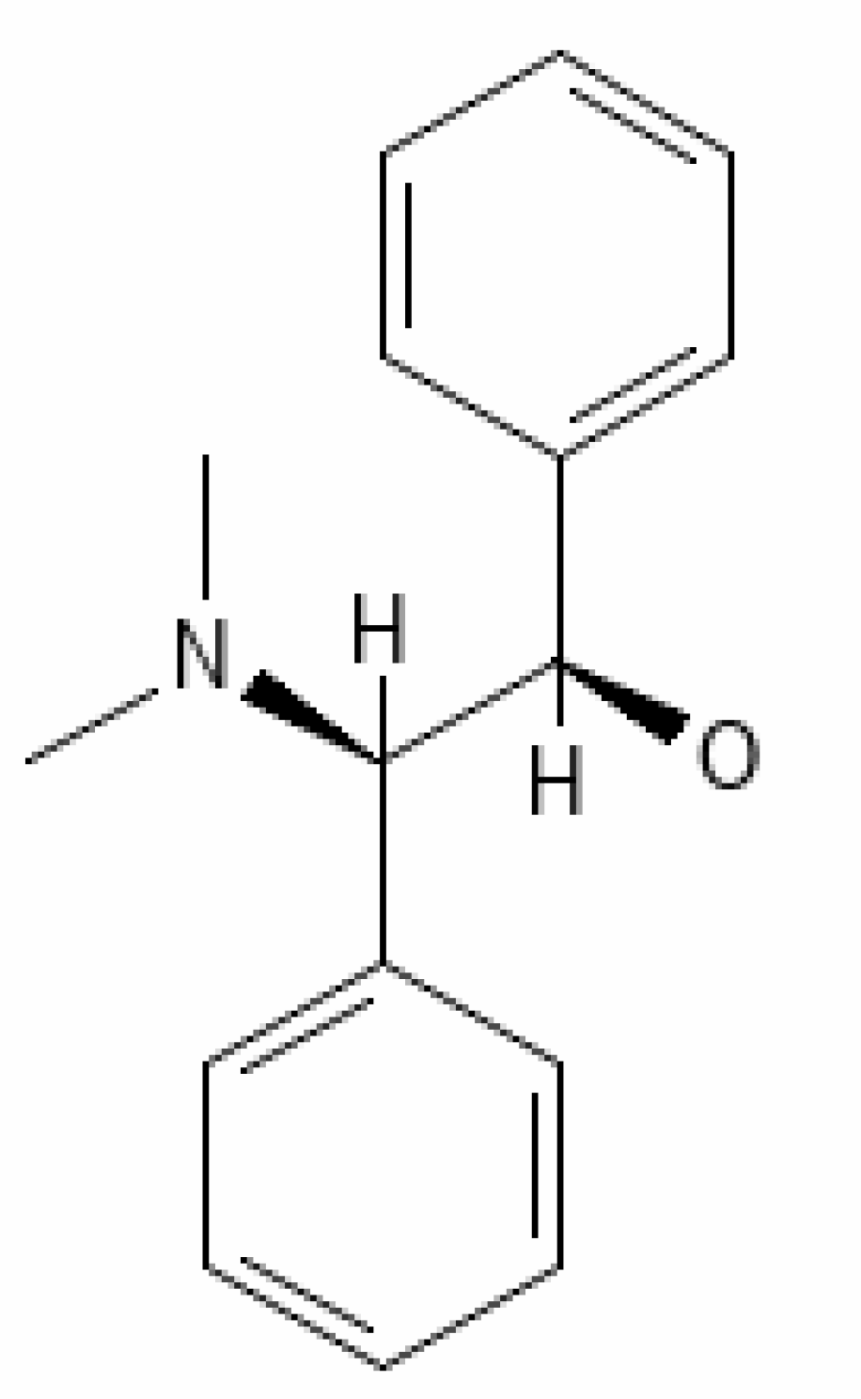	Methanolic	Turrini et al. (2016); Balkrishna et al. (2021)
18.	Withasomniferol A	101710595	-3.35	486.6	Leaves and Roots	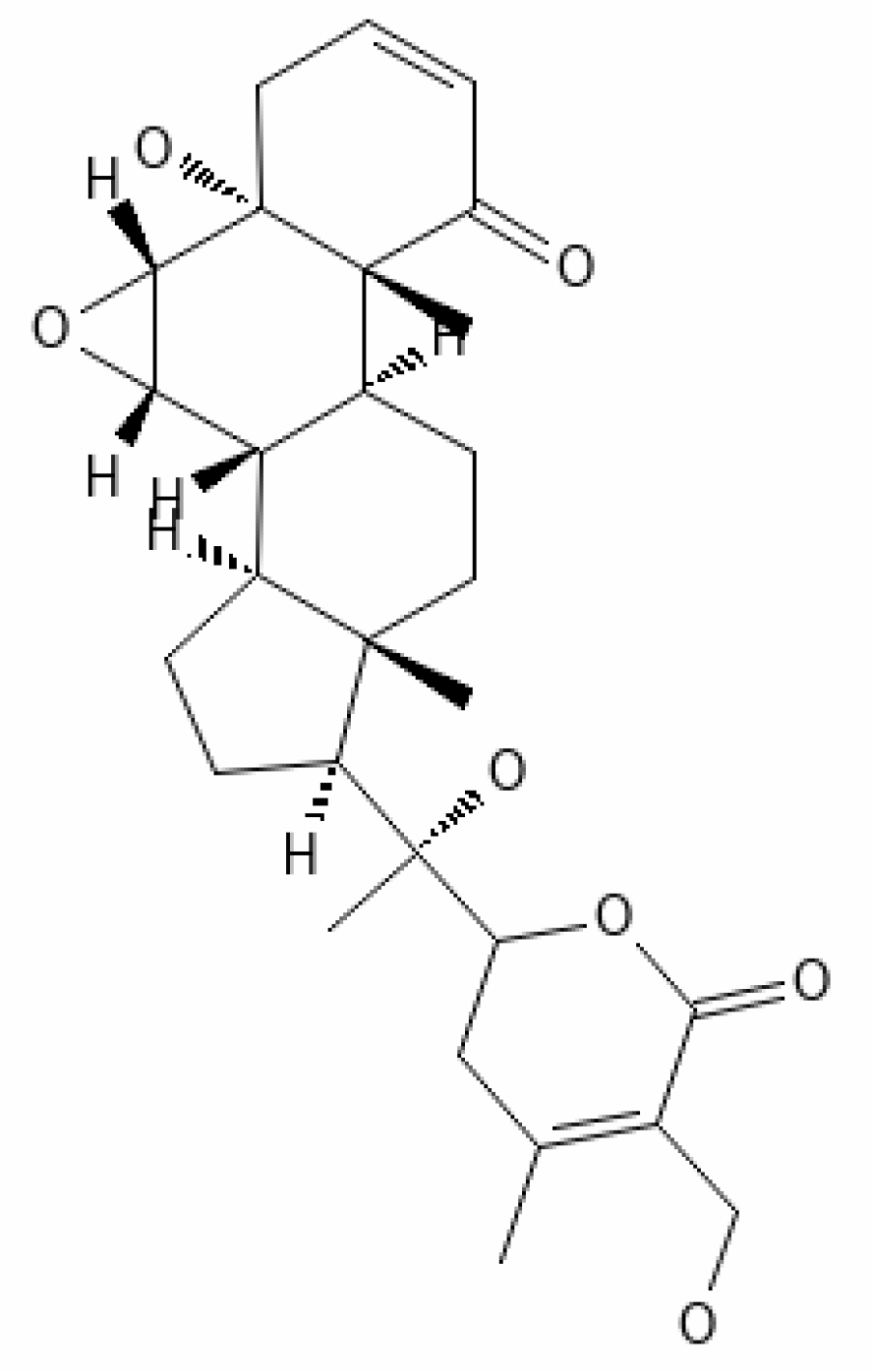	Methanolic	Singh et al. (2020)
19.	Withasomniferol B	101710596	-4.22	472.6	Leaves and Roots	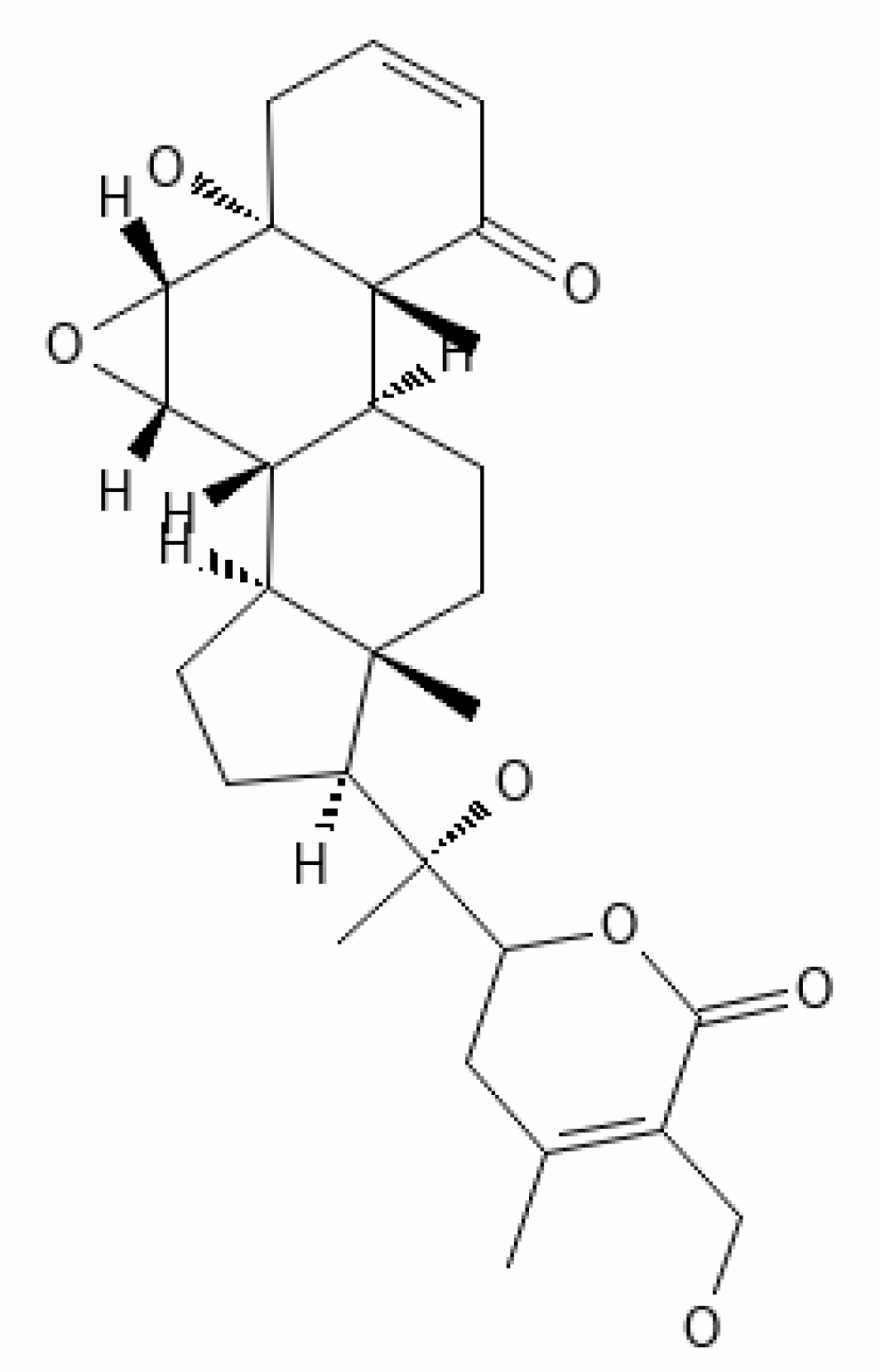	Methanolic	Singh et al. (2020)
20.	Withasomniferol C	101710597	-4.88	470.6	Leaves and Roots	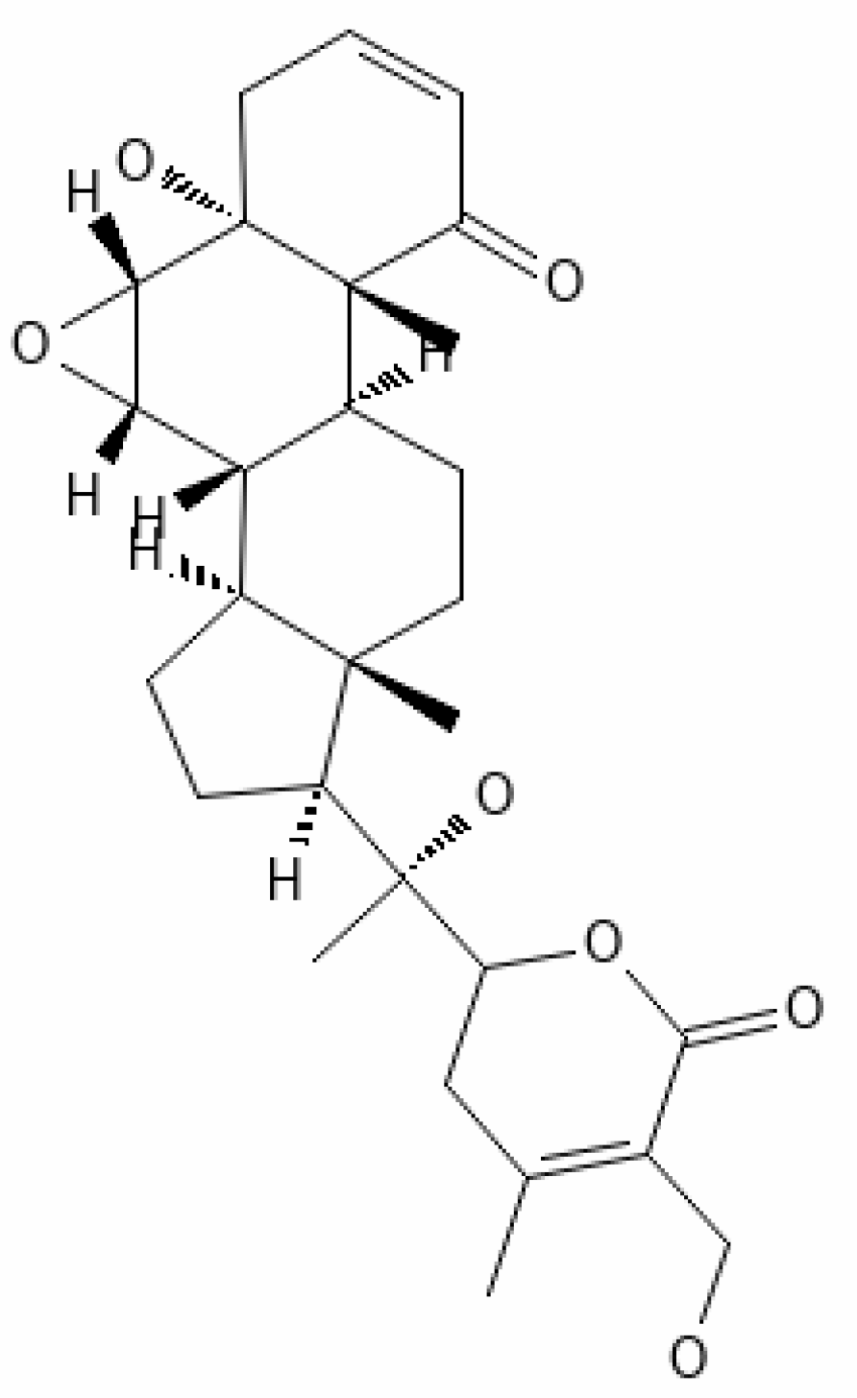	Methanolic	Kandagalla et al. (2022)
21.	β-sitosterol	348285530	-5.44	414.71	Leaves and Roots	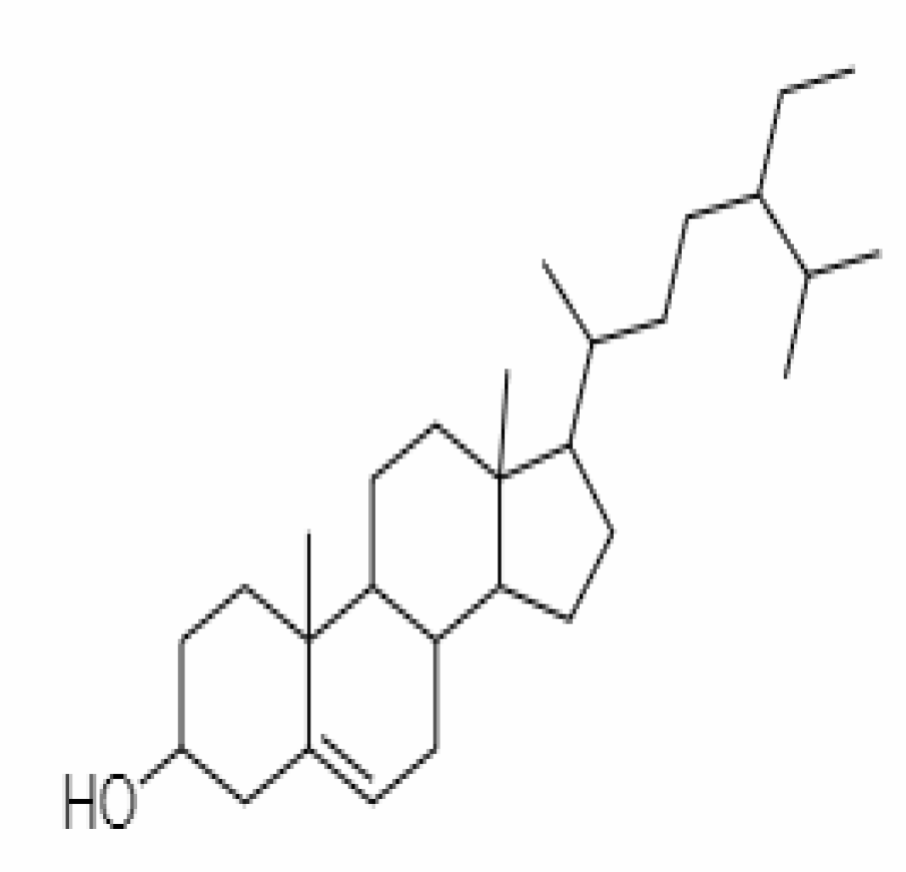	Methanolic	Kandagalla et al. (2022)
22.	Withanolide	53477765	-5.26	470.6	Leaves and Roots	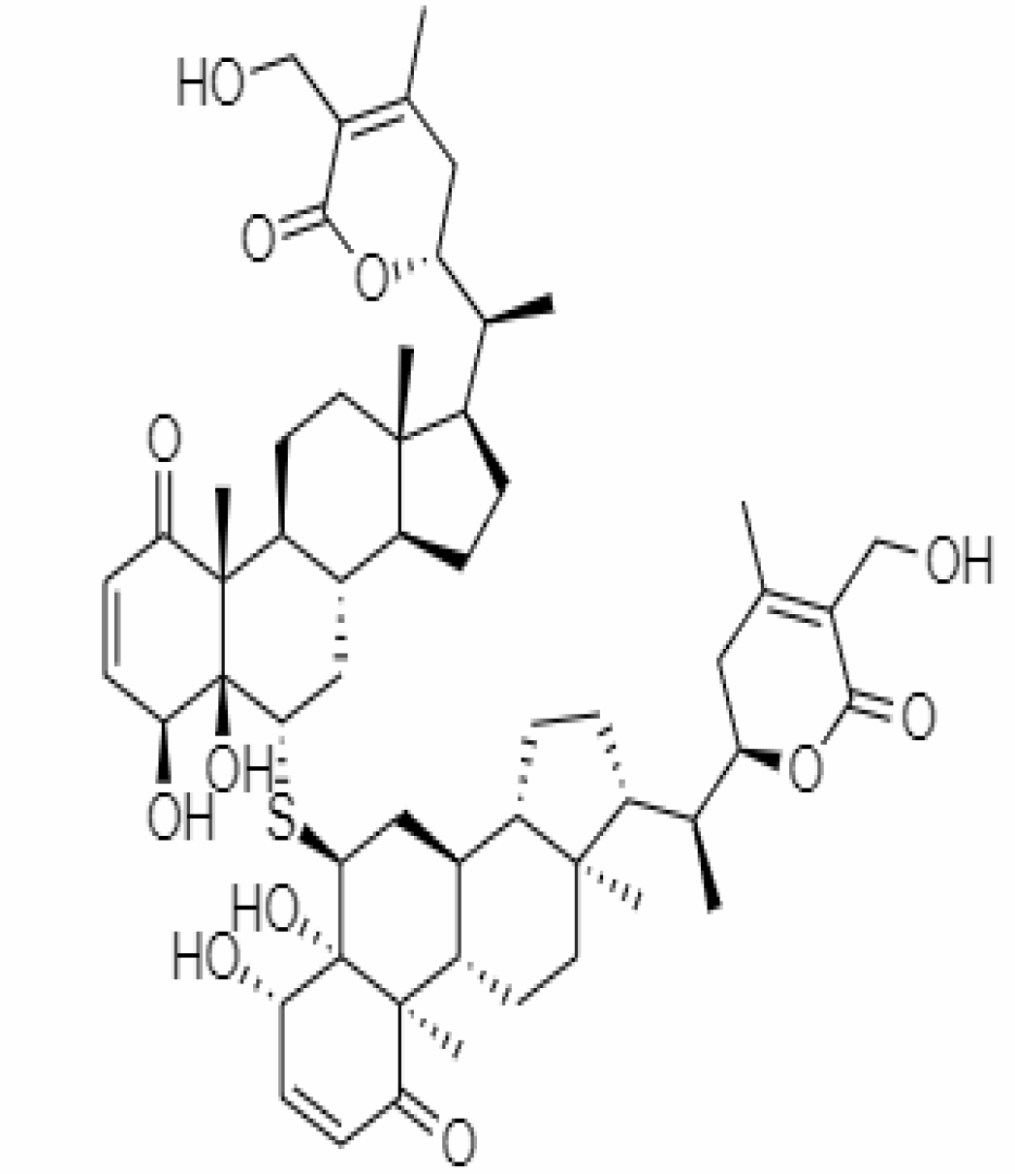	Methanolic	Kalra and Kandimalla (2021)
23.	Withanosides II	101168811	-11.30	798.9	Leaves and Roots	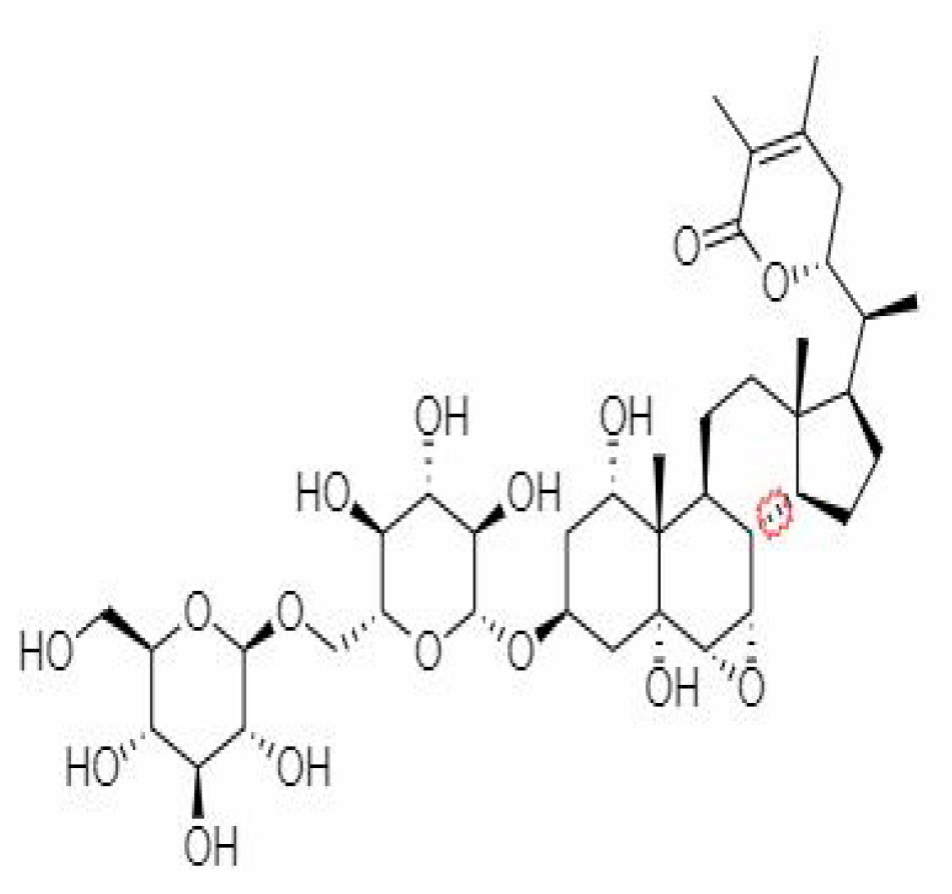	Methanolic	Kalra and Kandimalla (2021)
24.	Withanosides III	101168810		652.8	Leaves and Roots	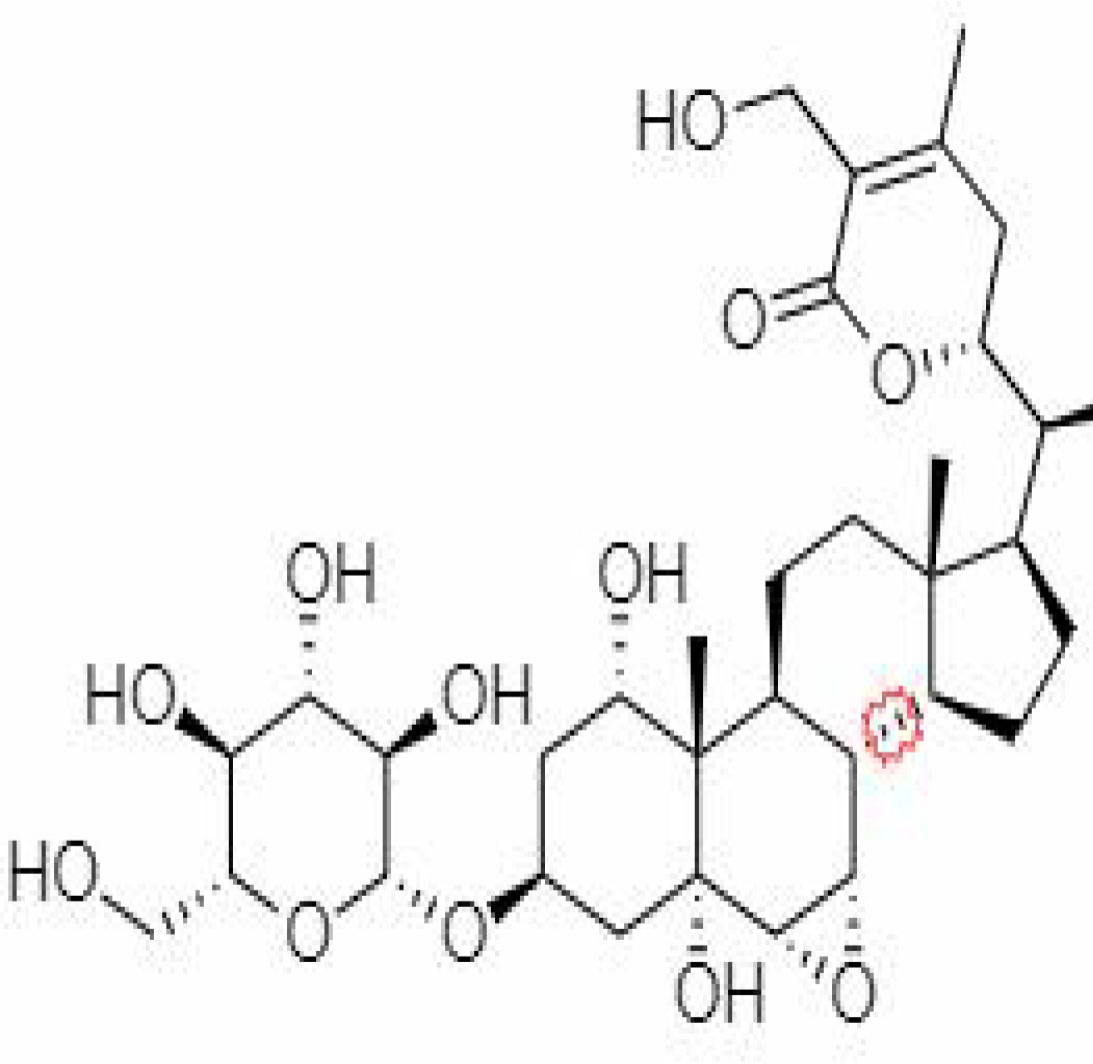	Methanolic	Ali et al. (1997); Jayaprakasam et al. (2004); Kalra and Kandimalla (2021)
25.	Withanosides IV	71312551	-11.02	782.9	Leaves and Roots	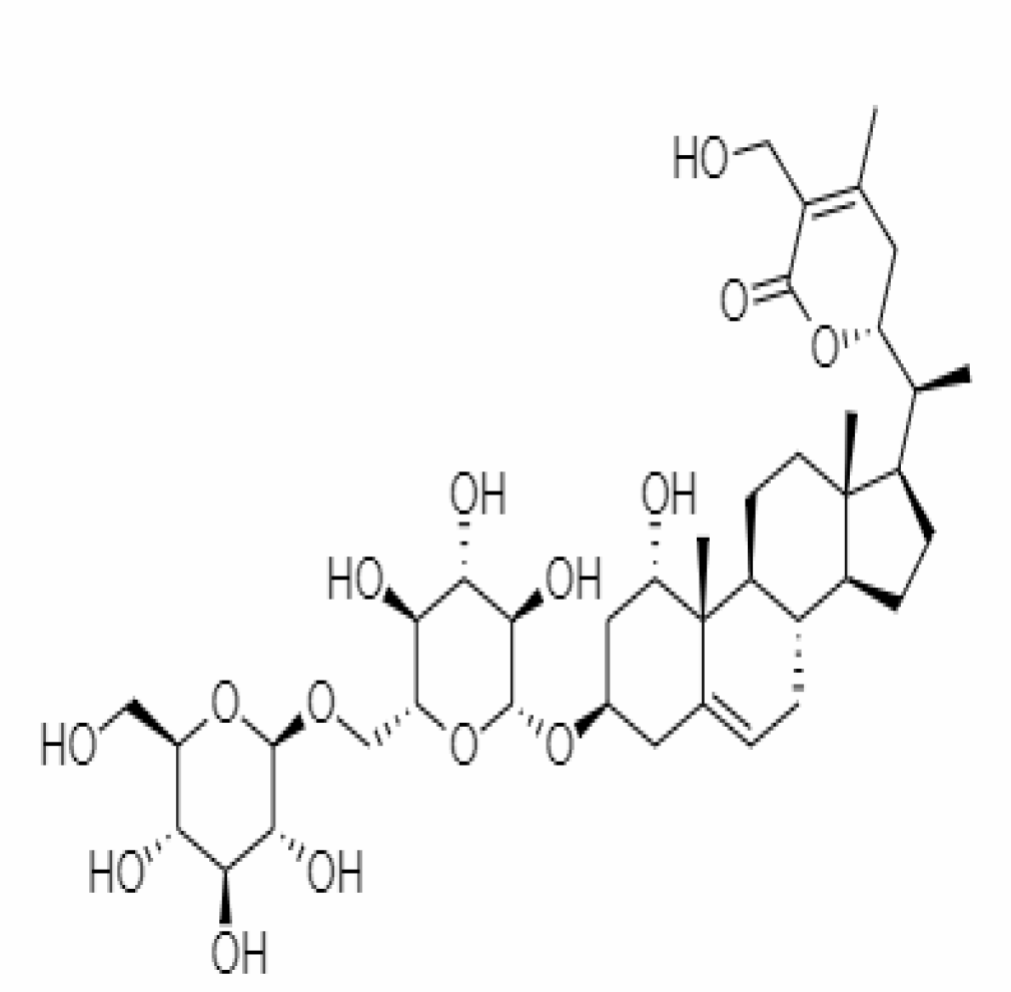	Methanolic	Kalra and Kandimalla (2021)
26.	Withanosides V	10700345	-8.96	766.9	Leaves and Roots	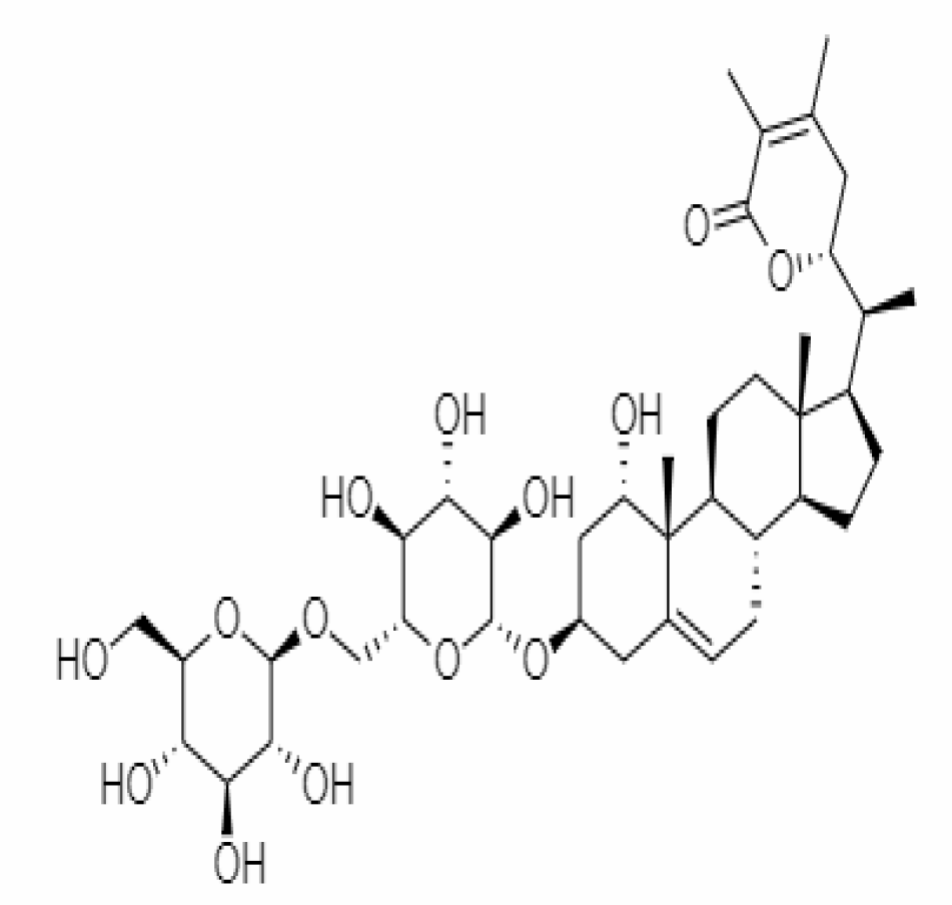	Methanolic	Kalra and Kandimalla (2021)
27.	Withanosides VI	91827019		782.9	Leaves and Roots	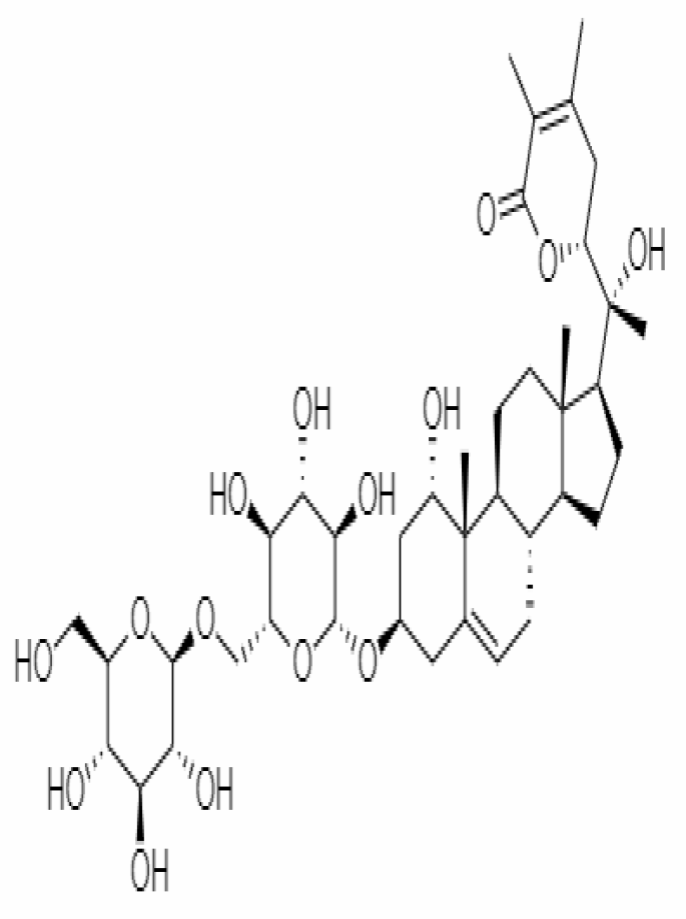	Methanolic	Kalra and Kandimalla (2021)
28.	Physagulin D	10100412	-4.48	620.8	Leaves and Roots	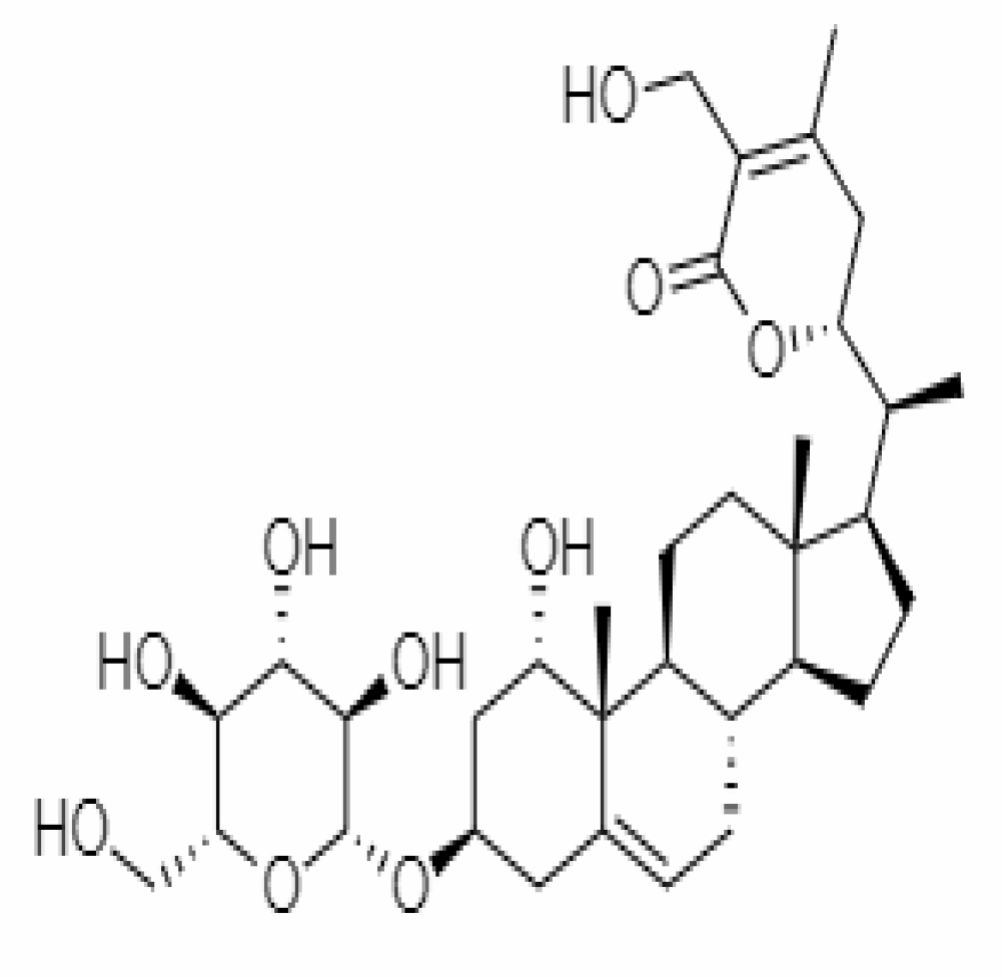	Methanolic	Kumar and Singh (2020)
29.	Withasomnilide	102066413	-4.98	470.6	Stem Bark	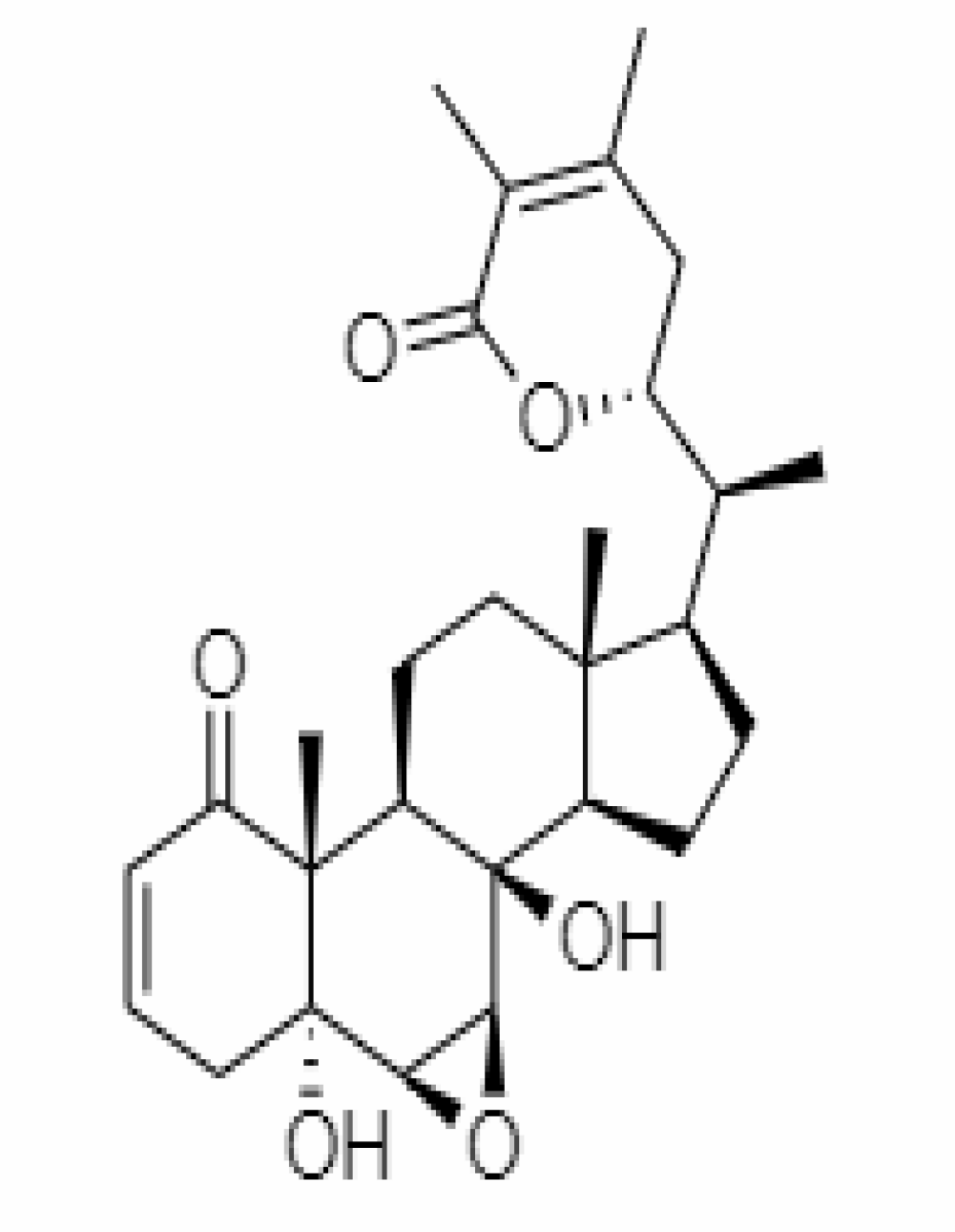	Ethanolic	Tong et al. (2011)
30.	Somniferanolide	102066415	-7.89	468.6	Stem Bark	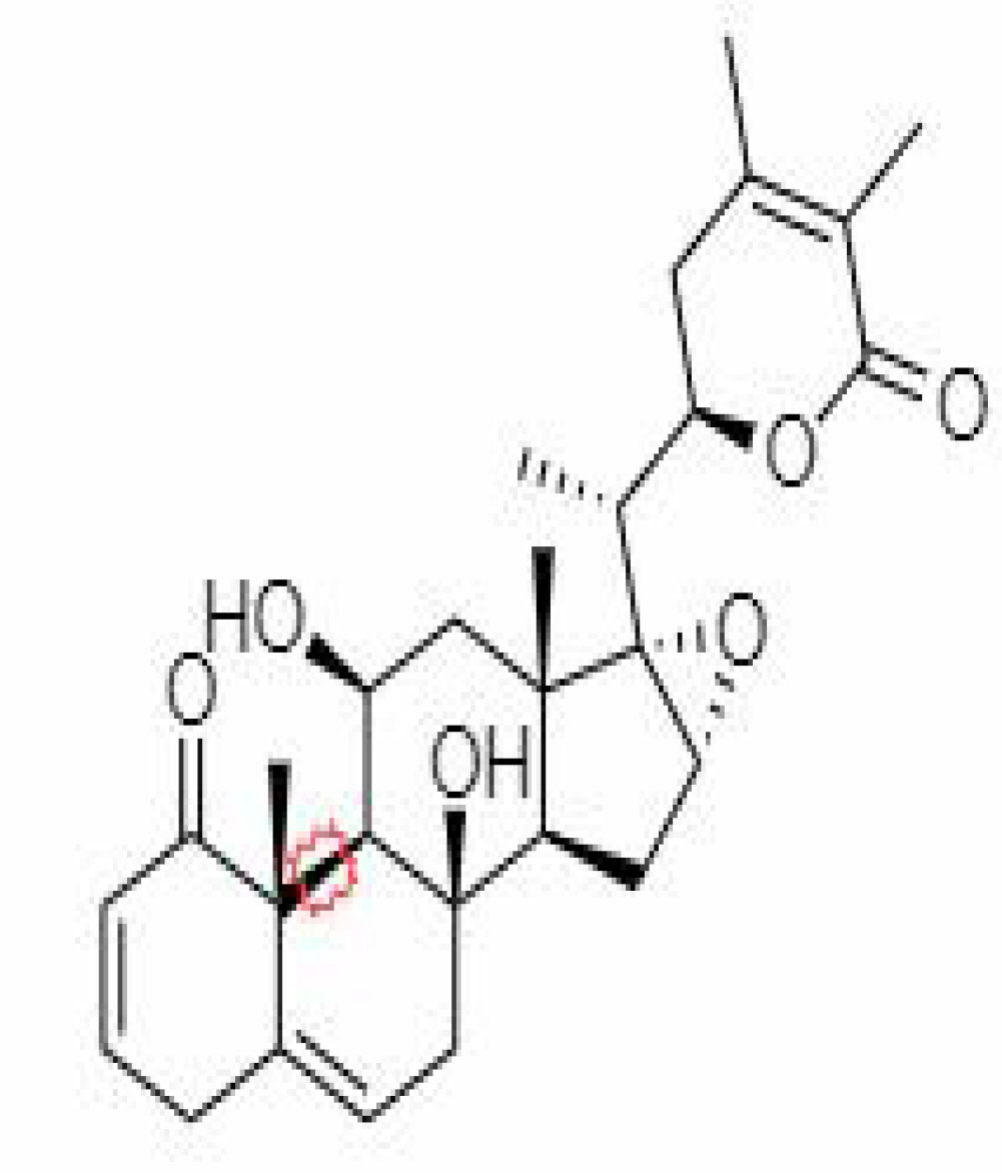	Methanolic	Hasan et al, 2022; Ali et al, 1997
31.	Somniferine	14106343	9.62	608.7	Stem Bark	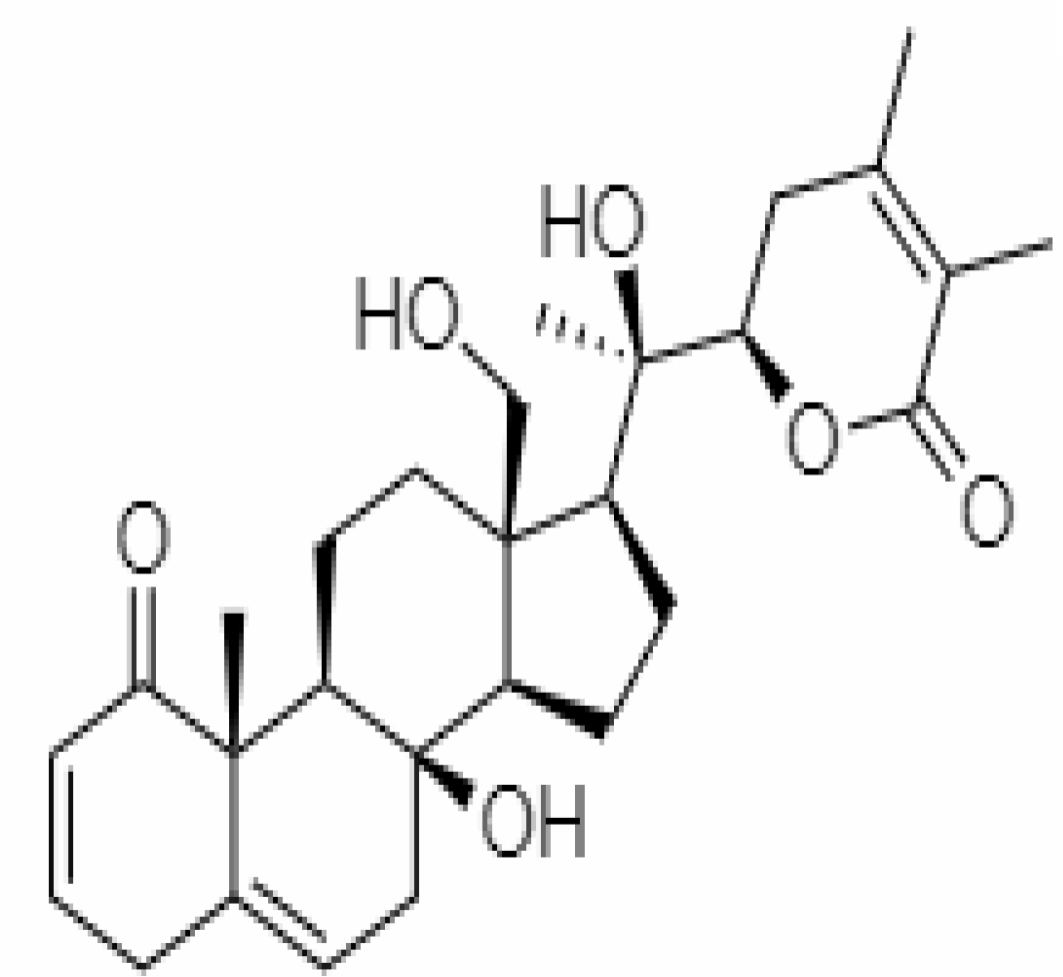	Methanolic	Hasan et al, 2022; Ali et al, 1997
32.	Withasomniferanolide	102066414	-3.53	470.6	Stem Bark	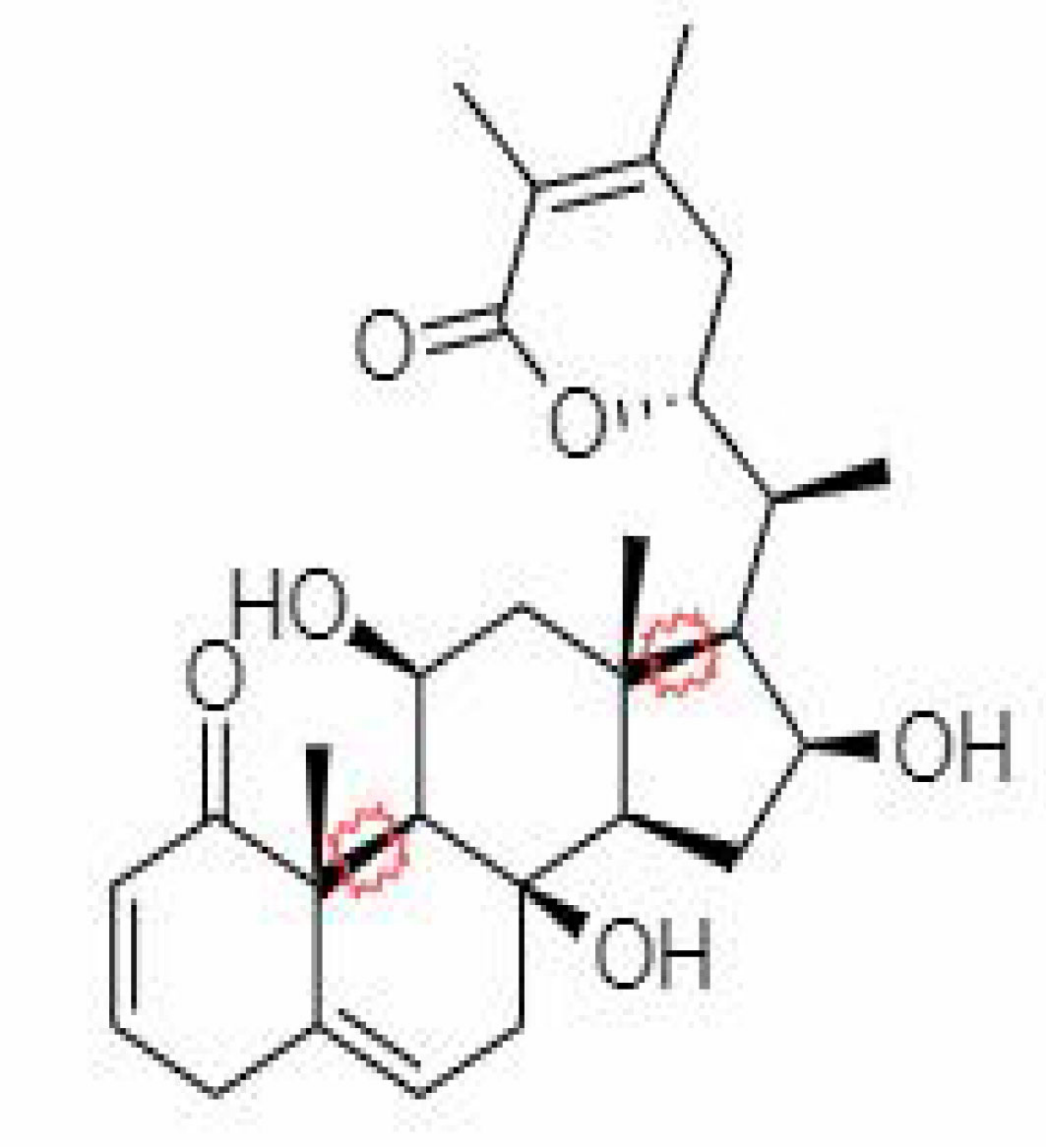	Methanolic	Kalra and Kandimalla (2021)
33.	Somniwithanolide	102066417	-5.42	486.6	Stem Bark	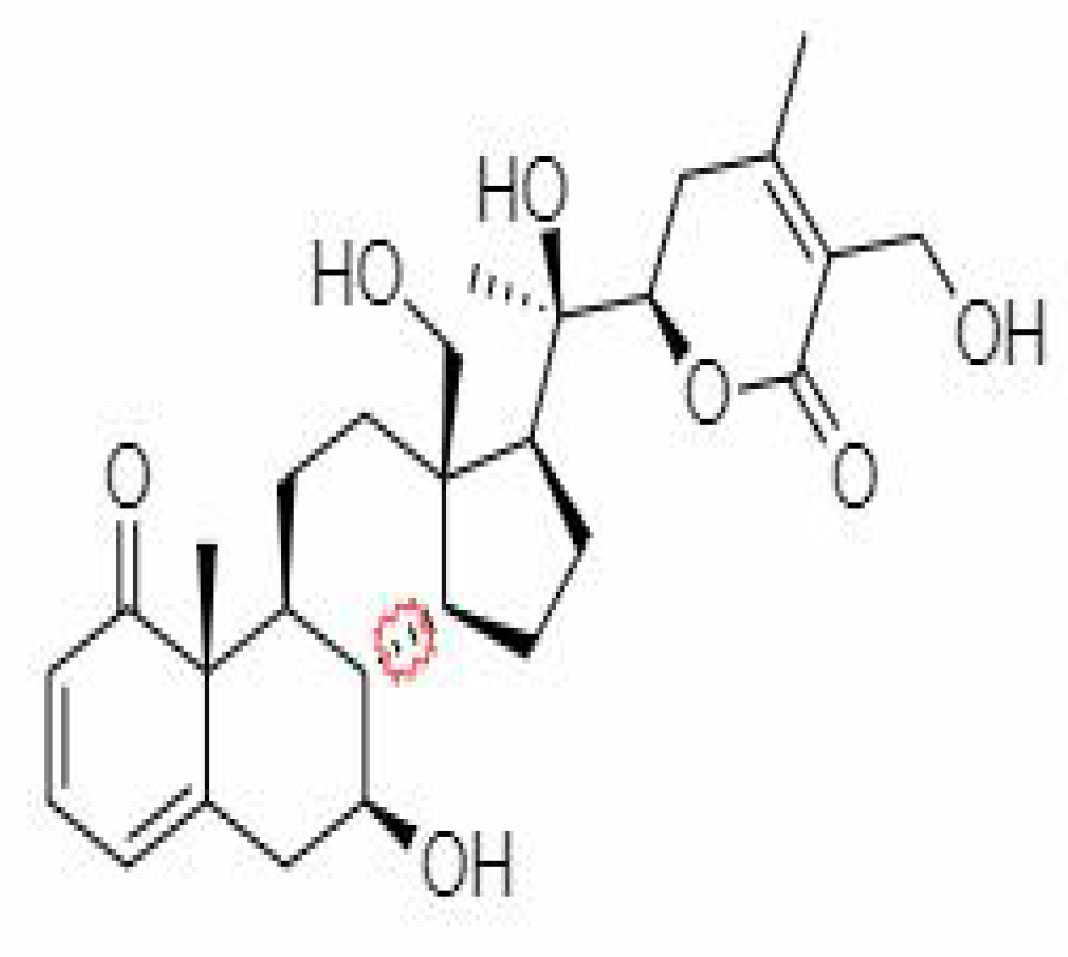	Methanolic	Prajapati et al. (2022)
34.	Withanolide	9469353	-5.81	370.4	Whole plant	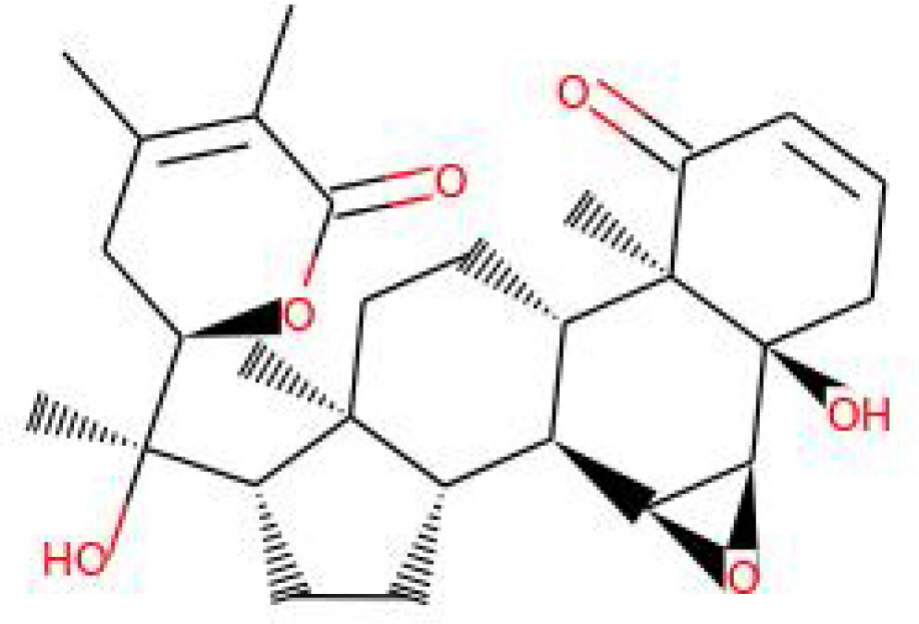	Methanolic	Kirson et al. (1971); Kirson et al. (1971); Matsuda et al. (2001); Jayaprakasam and Nair (2003); Choudhary et al. (2010)
35.	Viscosalactone B	57403080	-11.1	488.6	Whole plant	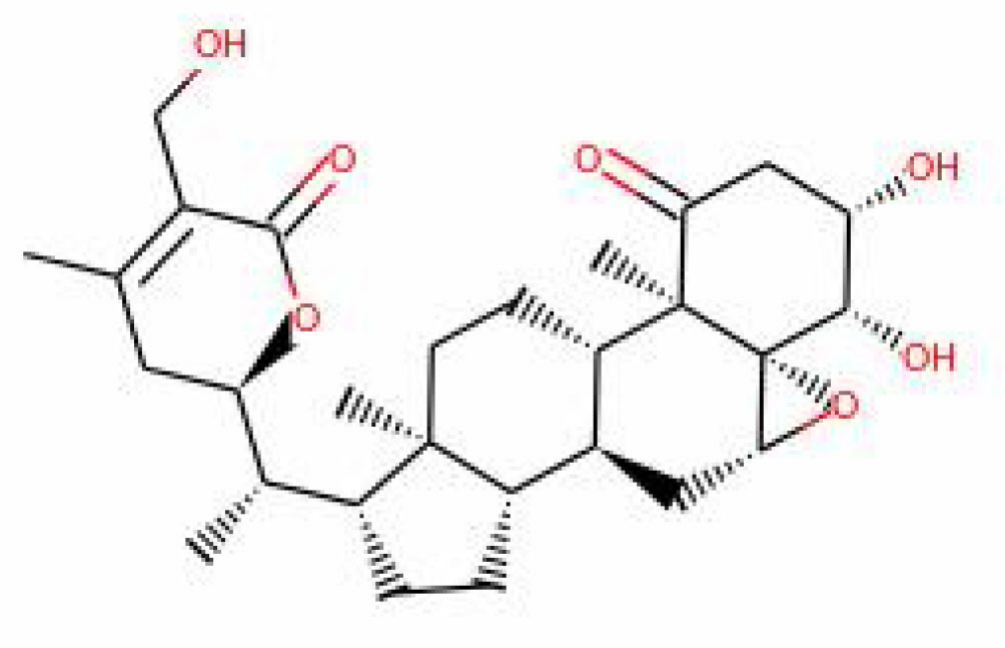	Methanolic	Borse et al., 2020
36.	27-acetoxy -4β,6α –dihydroxy -5β-chloro -1-oxowitha -2,24-dienolide	21044792	-3.30	523.34	Aerial parts	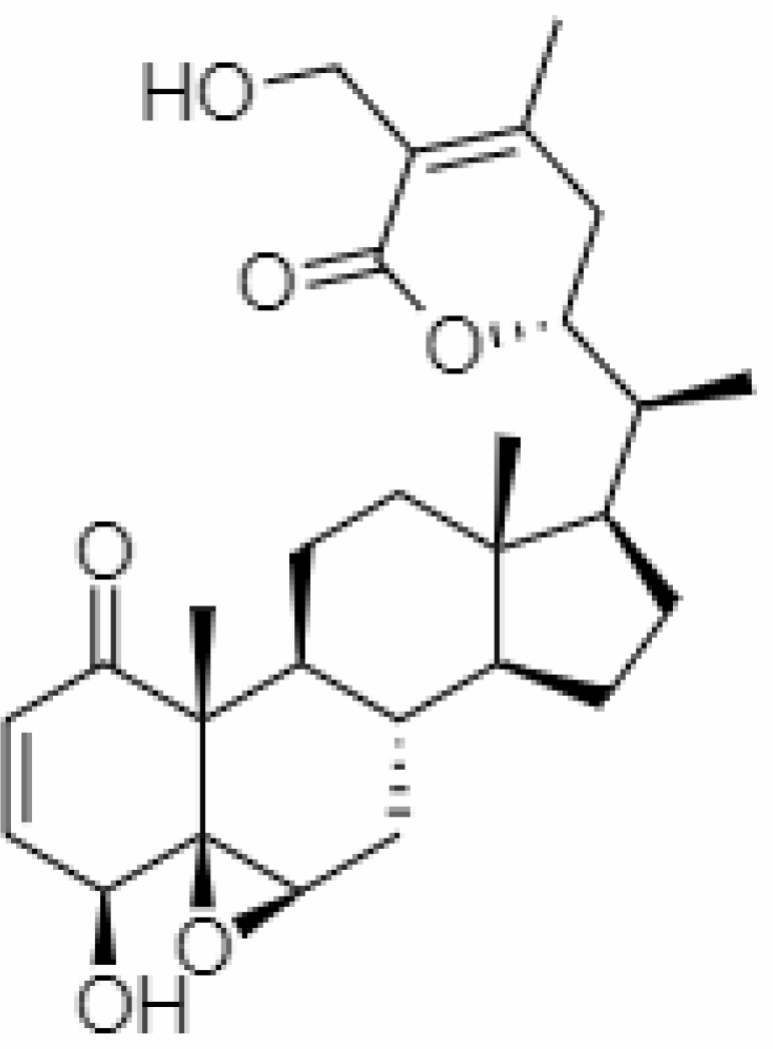	Methanolic	Kuboyama et al. (2006); Tong et al. (2011); Seth et al. (2020)

## 7 Possible roles of *Withania somnifera* as immune homeostasis/therapeutics in the management of COVID-19

During the viral infection such as SARS-CoV-2, it has been seen that the secretions of inflammatory molecules at higher risk with elevated levels of (macrophages and neutrophils accompanied by lower levels of T cells and B cells) (Sadanandam et al., 2020). In this approach, a multicentric clinical investigation has been set up by the Ministry of AYUSH, Government of India to mitigate the deleterious effect of inflammatory cytokines in the management of COVID-19. Several studies have shown that administration of *Withania somnifera* solutions enhanced the immune system with the proliferation of immune response such as activation of T cells and B cells, platelets, and NK cells accompanied by maintaining the Th1/Th2 response in different immunocompromised/infected animal models (Kushwaha et al., 2012a; Sun, 2019). In addition to that, administration of extracts of Withaferin A and *Withania somnifera* have suppressed the inflammatory cytokines such as IL-1β, IL-6, and TNF-α and modulated many inflammatory pathways including NLRP3 inflammasome, NF-κB pathway, and infection-induced TLR4 expression in macrophages through chaperone activity of peroxiredoxins as interventions of inflammatory response in different studies models (Noh et al., 2016; Khan et al., 2018; Wang et al., 2020b; Balkrishna et al., 2020; Aziz et al., 2020). Thus, *Withania somnifera* will be the potential herbal medicine to enhance immune system as a modulator of high-risk inflammatory response of COVID-19 infection (Parwe et al., 2020; Varma et al., 2020).

On the other hand, it has been demonstrated that systemic inflammatory response along with the COVID-19 infection resulted in multi-organ failure due to the releasing of inflammatory mediators in the blood of several organ and this amplify the complication of the affected organs. In this regard, *Withania somnifera* has emerged as a potential candidate to protect organs against the side effects caused due to the inflammatory reactions of COVID-19. Several studies have reported that the extracts of *Withania somnifera* containing Withanolide A show neuroprotective candidate against hypoxia-induced and promote cerebral ischemia-induced apoptosis for curing the neurological disorders (Kuboyama et al., 2005; Mukherjee et al., 2020). Likewise, extracts of *Withania somnifera* have shown wonderful response in maintaining elevated levels of cardiac troponin I and IL-6 in the cardiomyopathy condition during the infection (Adão and Guzik, 2020; Ingawale et al., 2020). They have also shown the modulator of proliferating cell nuclear antigen (PCNA) by regulating the NF-κB transcription factor against the pulmonary inflammation (Kaur et al., 2015b). In addition, extracts of *Withania somnifera* were found to restore the antioxidant enzyme expression in GI, kidney cells, and pancreas and to reduce the serum levels of urea and creatinine in kidney and muscles during the viral infections in the different study models. Withaferin A from *Withania somnifera* protects the cytotoxicity effect of bromobenzene in the liver and kidney cells (Jeyanthi and Subramanian, 2010; Vedi and Sabina, 2016; Tiruveedi et al., 2018). Thus, it can be proposed that extract of *Withania somnifera* can be the potential drug against the biological consequences of infections and inflammation and also decelerate the side effects in COVID-19 patients. The current therapeutic approaches in the management of COVID-19 are the use of several repurposing drugs such as chloroquine, azithromycin, remdesivir, and hydroxychloroquine. However, the beneficial effects of all the drugs in the therapy of COVID-19 are still inconclusive (Ferner et al., 2020). Several reports have shown that use of such repurposing drug might accelerate the conditions of heart and respiratory failure in the patients of COVID-19 (Lane et al., 2020). In such condition, *Withania somnifera* is acting as a miracle drug for cardiorespiratory protection and enhances the antibody titer in the immunocompromised model and thus acts as a synergistic effects in the management of COVID-19 (Davis and Kuttan, 2000; Davis and Kuttan, 2002). To circumvent the pandemic of COVID-19, there is an urgent need for effective vaccine and the efficacy of the vaccine can be enhanced by the co-formulation of the mixture of vaccine and herbal immunostimulants such as the extract of *Withania somnifera*, and this formula will lead to an additive adjuvant to boost vaccine immunogenicity. In this aspect, extract of *Withania somnifera* has been shown to recruit more NK cells against influenza infection, which led to the elimination of toxic cells in the infected model (Davis and Kuttan, 2002). All these previously beneficial effects of *Withania somnifera* prove that it will be the leading candidate to explore the vaccine development in the management of COVID-19.

## 8 Conclusion

We have tried to update the current knowledge and information of SARS-CoV-2 infection, mode of transmission, clinical symptoms diagnosis, and possible treatments through different approaches. With the era of different harmful after-effects from re-purposed drugs, we are now moving forward to find the herbal cure for the SARS-CoV-2 virus disease using traditional knowledge systems along with modern medicine. With this reference, the first choice may be *Withania somnifera* cocktail with other herbal medicines for better efficiency. We explored the various bioactive compounds of *Withania somnifera*, which have the potential to inhibit the interaction between RBD of SARS-CoV-2 S-protein and ACE2 receptor. Because the interaction of RBD of SARS-CoV-2 S-protein and ACE2 receptor is very important for the entry of virus into the host cells during infection, thus bioactive compound of *Withania somnifera* such as withanone and withaferin-A may be implicated for the management and treatment of COVID-19 (CMP, 2020). However, advanced research is required to understand molecular mechanism of bioactive compounds of *Withania somnifera* for improvement in clinical outcome of patients with COVID-19 (CMP, 2020). A deeper multidimensional understanding of *Withania somnifera*’s biological mechanisms will be an encouraging field for future research.

## Author contributions

Conceptualization: MS, KJ, and DS; methodology: SB, MA, FK, and NKP; validation: AC, MA, AS, and MS; formal analysis: SB, and NKP; investigation, AC, FK, MA, AS, and data curation, FK, SK, MS, SB, and MA; writing—original draft preparation: MS, DS, and SK; writing—review and editing: DL, MA, SUDK, and SB; visualization: AS, AC, MA, and NKP; supervision: SK and DL; project administration: MA. All authors contributed to the article and approved the submitted version.

## Funding

This work was supported by the Sichuan Science and Technology Program (No. 2021YFH0093) and the China Postdoctoral Science Foundation (No. 2020M673187).

## Conflict of interest

The authors declare that the research was conducted in the absence of any commercial or financial relationships that could be construed as a potential conflict of interest.

## Publisher’s note

All claims expressed in this article are solely those of the authors and do not necessarily represent those of their affiliated organizations, or those of the publisher, the editors and the reviewers. Any product that may be evaluated in this article, or claim that may be made by its manufacturer, is not guaranteed or endorsed by the publisher.

## Abbreviations

SARS-CoV-2, Severe acute respiratory syndrome coronavirus 2; CoV, Coronavirus; WHO, World Health Organization; RNA, Ribonucleic acid; ORF1, Open reading frame 1; ACE2, Angiotensin-converting enzyme 2; GI, Gastrointestinal; SARS, Severe acute respiratory syndrome; IFN, Interferon; IgG, Immunoglobulin G; JAK, Janus kinase; TMPRSS2, Transmembrane protease serine 2; CMK, Chloromethyl ketone; FDA, Food and Drug Administration; RdRp, RNA-dependent RNA polymerases; HIV, Human immunodeficiency virus; NK cell, Natural killer cell; PCNA, Proliferating cell nuclear antigen; GABA, Gamma-aminobutyric acid.
